# Long-term effectiveness of app-based therapy for female urinary incontinence: 48-week follow-up of a randomised controlled trial

**DOI:** 10.1016/j.eclinm.2026.104298

**Published:** 2026-10-28

**Authors:** Lisa Frey, Axel Haferkamp, Walter Lehmacher, Gregor Duwe, Jan Hendrik Börner, Kerstin A. Brocker, Leonie Ratz, Diana Moldovan, Jan Erik Krieger, Kurt Miller, Laura Wiemer, C Patrick Papp

**Affiliations:** a Department of Urology and Pediatric Urology, University Medical Center of the Johannes Gutenberg-University Mainz, Mainz, Germany; b Institute of Medical Statistics and Computational Biology, University of Cologne, Cologne, Germany; c Practice for Gynecology and Obstetrics, Karlsruhe, Germany; d Practice for Urology, Berlin, Germany; e Department of Clinical Research, Kranus Health, Munich, Germany; f Department of Urology, Charité–Universitätsmedizin Berlin, Corporate Member of Freie Universität Berlin, Humboldt-Universität zu Berlin and Berlin Institute of Health, Berlin, Germany

**Keywords:** Urinary incontinence, Digital therapeutics, Mobile applications, Digital health application (DiGA), Long-term follow-up, Long-term data, Pelvic floor muscle training, Patient reported outcome measures

## Abstract

**Background:**

Long-term data for digital therapeutics in urinary incontinence are scarce. We assessed whether improvements with app-based multimodal therapy are sustained over 48 weeks, and outcomes after a 12-week delay in initiation.

**Methods:**

In this prespecified 48-week follow-up of the DINKS (Digital Incontinence Study) randomised waitlist-controlled trial, women (≥18 years) with stress, urge, or mixed urinary incontinence recruited between April 30 and September 2 2024 across Germany received app-based therapy immediately or after a 12-week waitlist, alongside standard care. The primary outcome was change from baseline in ICIQ-UI-SF (International Consultation on Incontinence Questionnaire–Urinary Incontinence Short Form) score at weeks 12, 24, 36, and 48. Trial registration: NCT06389838.

**Findings:**

Data availability declined progressively from 186 of 194 participants (95.9%) at week 12–66 (34.0%) at week 48. Pooled improvements exceeded the minimal clinically important difference at 12, 24, and 36 weeks after therapy start (ICIQ-UI-SF at 36 weeks −4.13, 95% Confidence Interval (CI) −4.80 to −3.47), and 73–77% of participants met the responder threshold. Differences between immediate and delayed initiation were small (ICIQ-UI-SF at 36 weeks 0.08, −1.09 to 1.25). The week-48 estimate (−3.88, −4.74 to −3.03) was intervention-group only, from 27 of 96. No adverse events were reported.

**Interpretation:**

App-based multimodal therapy was associated with clinically meaningful improvements sustained through at least 36 weeks after intervention start. Differences between immediate and 12-week-delayed initiation were small relative to the minimal important difference, but no non-inferiority margin was prespecified and only one delay was tested; symptoms were unchanged while waiting, so waiting times should still be minimised. Confirmation in larger trials with active comparators is needed.

**Funding:**

Kranus Health GmbH.

**Research in context**

**Evidence before this study**

We searched PubMed, Embase, and the Cochrane Library from inception to Dec 31, 2025, using combinations of controlled vocabulary and free-text terms related to urinary incontinence, digital or app-based interventions, pelvic floor muscle training, and long-term outcomes. Previous studies demonstrated that digital interventions can improve urinary incontinence symptoms. Early evidence focused primarily on pelvic floor muscle training for stress urinary incontinence, with sustained benefits reported for up to 24 months. More recently, multimodal app-based interventions incorporating pelvic floor muscle training, bladder training, and lifestyle guidance have shown effectiveness in women with stress, urgency, and mixed urinary incontinence, including sustained effects over one year, though these were evaluated in primary care against usual care rather than over extended follow-up as a primary aim. However, important evidence gaps remain, including the lack of longer-term follow-up for multimodal digital therapeutics that has undergone national regulatory approval beyond medical-device certification, the absence of objective usage–response analyses based on backend usage data, and no evaluation of delayed versus immediate access. Additionally, fewer than 50% of affected women seek professional care, with stigma and embarrassment identified as primary barriers, underscoring the need for accessible, privacy-preserving interventions.

**Added value of this study**

This prespecified 48-week follow-up provides the longest reported assessment, to our knowledge, of a certified multimodal digital therapeutic that has undergone national regulatory approval evaluated across all incontinence subtypes, and the first to examine delayed versus immediate access and objective usage–response relationships. The programme integrated bladder training, cognitive behavioural strategies, mindfulness exercises, and lifestyle guidance, unlike previous interventions limited primarily to pelvic floor exercises. Key novel findings include: after a single fixed 12-week delay, the control group reached improvements of similar magnitude once therapy began, although no non-inferiority margin was prespecified; exploratory analyses of objective backend usage data reveal no usage-response relationship, suggesting a possible threshold-based effectiveness; and 72–79% of participants maintained clinically meaningful response across follow-up, with 65% of participants with available data continuing independent exercises without app support at week 48; estimates become progressively less certain over time, and the 48-week timepoint is exploratory and intervention-group only. The privacy and accessibility of app-based treatment are particularly relevant for addressing stigma-related barriers to care-seeking.

**Implications of all the available evidence**

Our findings extend the evidence base by showing durable long-term effectiveness of multimodal app-based therapy across urinary incontinence subtypes. The absence of a clear usage–response relationship suggests that behaviour change initiation may matter more than engagement intensity, potentially reducing patient burden and improving adoption. From a health systems perspective, sustained benefits without continuous active treatment support cost-effective implementation, with potential to reduce progression to surgery and reliance on incontinence products. Future research should identify predictors of treatment response, compare different delay durations, evaluate cost-effectiveness, develop strategies for non-responders, and explore generalisability across diverse populations.

## Introduction

Urinary incontinence affects an estimated 25–45% of adult women globally,^1^ with approximately 55–60 million Europeans currently living with continence health problems.^2^ Beyond impacts on quality of life and psychological wellbeing, the condition carries substantial economic burden (€69.1 billion in the European Union in 2023, projected to reach €86.7 billion by 2030) and environmental impact (carbon emissions from incontinence products projected to reach 1.1 billion kg CO_2_e (carbon dioxide equivalent) annually by 2030).^2^

Clinical practice guidelines consistently recommend conservative treatments, particularly pelvic floor muscle training and lifestyle modifications as first-line therapy for all urinary incontinence subtypes.^3^, ^4^ However, implementation remains suboptimal due to limited specialist access, long waiting times,^2^ and patient barriers including stigma and underreporting.^5^ Fewer than 50% of affected individuals initiate professional care within two years of symptom onset; the majority delay their first healthcare engagement for approximately three to six years, with a significant proportion waiting more than a decade.^6^

Digital therapeutics offer scalable delivery of evidence-based interventions. In Germany, digital health applications (Digitale Gesundheitsanwendungen; DiGAs) are rigorously regulated medical devices requiring demonstrated clinical benefit through randomised controlled trials and meeting stringent safety and data protection requirements.^7^ Once certified, DiGAs can be prescribed and reimbursed by statutory health insurance.

Given substantial workforce shortages (an estimated 1 million health workers shortage in Europe)^2^ and growing demand from an ageing population, digital interventions represent a critical strategy to bridge treatment gaps. Recognition of this need has catalysed multiple international awareness and policy initiatives, including Europe’s “An Urge to Act” campaign,^2^ the United States’ CDC (Centers for Disease Control and Prevention)-funded “We Count” campaign,^8^ and Australia’s National Continence Program,^5^ all emphasising the urgent need for scalable, evidence-based solutions.

The initial 12-week results of the Digital Incontinence Study (DINKS) demonstrated that app-based therapy combining pelvic floor training, bladder training, cognitive behavioural strategies, and lifestyle guidance significantly reduced incontinence episode frequency (mean relative reduction 60.95%, 95% CI −71.7 to −46.7; p < 0.001) and improved symptom severity as measured by the ICIQ-UI-SF (International Consultation on Incontinence Questionnaire–Urinary Incontinence Short Form) (mean difference −3.94 points, 95% CI −4.74 to −3.14; p < 0.001) compared with usual care alone.^9^ Additionally, the intervention reduced pad usage by 2.63 pads per day (relative decrease 27.8%), with a between-group treatment effect of 2.6 pads (95% CI 1.6–3.5; p < 0.0001).^9^

Previous follow-up studies demonstrated clinically meaningful symptom improvement but differ from the present programme in scope and design. One app addressed stress incontinence only,^10^ and a separate app subsequently addressed urgency and mixed incontinence with a multimodal programme.^11^ Another multimodal programme included all subtypes, with effectiveness maintained at one year, but was evaluated in primary care under a non-inferiority design against usual care.^12^ Evidence therefore remains limited for a multimodal digital therapeutic that has undergone national regulatory approval, beyond medical-device certification alone, evaluated across all subtypes with respect to durability, delayed versus immediate access, and objective usage–response relationships.

We conducted a prespecified 48-week follow-up of the DINKS (Digital Incontinence Study) trial which included data from the early app group and from the late app group (waiting group) at baseline, week 12, 24, 36, and 48 to address these evidence gaps. We hypothesised that improvements achieved with app-based therapy would be maintained for at least 48 weeks. As a descriptive objective, we estimated the difference in outcomes between immediate and 12-week-delayed initiation at equivalent post-intervention intervals; no non-inferiority margin was prespecified.

## Methods

### Study design and participants

DINKS was a single-blind, waitlist-controlled, randomised trial, reported in accordance with Consolidated Standards of Reporting Trials (CONSORT) guidelines, as previously reported.^9^ Briefly, participants with stress, urge, or mixed urinary incontinence were recruited between April 30 and September 2, 2024, by 239 office urologists and gynaecologists from all regions of Germany. Eligible participants were adults (aged ≥ 18 years) assigned female at birth with stress, urge, or mixed urinary incontinence as defined by their treating urologist or gynaecologist, with at least one urinary incontinence episode per day. Ownership of a smartphone or tablet was required for study participation. Participants were permitted to continue pre-existing therapies that had been initiated more than 4 weeks before enrolment, including bladder-specific or incontinence medications. The initiation of new therapies during the study was documented at the outcome assessment.

Race and ethnicity were not recorded, as their collection was not part of the primary trial protocol; country of birth was the only related variable available, and was previously reported.^9^

After the initial 12-week randomized controlled trial (RCT), the control group received access to the app-based programme and both groups were surveyed at 12-week intervals until they reached week 48. Initiation of new therapies were assessed at every time point. Data acquisition and management from baseline through week 48 were performed by an independent contract research organization CW-Research & Management (Munich, Germany) using electronic case report forms (eCRF) implemented in DACIMA (EvidentIQ, Hamburg, Germany) and questionnaire-based assessments administered via LimeSurvey (LimeSurvey GmbH, Hamburg, Germany). Participants received automated email reminders with the link to the eCRF. Data quality was ensured through built-in validation checks, regular monitoring, and audit trails, and access to the final dataset was restricted to authorised study personnel. All data handling complied with General Data Protection Regulation (GDPR) and institutional data protection requirements.

### Ethics and registration

The trial was registered at ClinicalTrials.gov (NCT06389838) and approved by the scientific ethics committee of the State Medical Association Rhineland-Palatinate (approval number 2024-17525-BO). All participants provided written informed consent via DocuSign software.

### Randomisation and masking

Participants were randomly assigned 1:1 to receive either immediate app-based therapy (intervention group) or a 12-week waitlist followed by intervention (control group), using permuted block randomisation with a block size of four, stratified by age category (≤45, 46–60, >60 years) and incontinence subtype (stress, urge, mixed). The allocation sequence was generated and implemented automatically by the electronic study system, so that investigators had no knowledge of or influence over upcoming allocations. Because the app-based therapy was delivered openly, participants could not be blinded to allocation. After randomisation, the investigators had no further contact with participants and did not know their allocation: all follow-up data acquisition was conducted independently by the contract research organisation. Full details are reported in the primary trial publication.^9^

### Intervention

Participants in the intervention group received access to Kranus Mictera, a BfArM (Federal Institute for Drugs and Medical Devices)-certified digital health application (DiGA). The app-delivered therapy comprised multiple evidence-based components: (1) bladder diary with personalised feedback; (2) pelvic floor and physiotherapy exercises with adaptive progression; (3) bladder training and cognitive behavioural therapy techniques; (4) mental exercises including mindfulness, progressive muscle relaxation, and stimulus control; (5) acute urge control function for real-time symptom management; (6) daily educational content tailored to individual needs and incontinence subtype; and (7) progress tracking with downloadable therapy reports for clinical consultations.

The therapy report integrates with the electronic patient record, enabling physicians to review symptom improvements, ICIQ-UI-SF score changes, and bladder diary patterns to inform data-driven treatment decisions. All content was evidence-based, guideline-based, and tailored to individual needs. The control group received usual care during the 12-week waitlist period, which consisted of participants continuing their current management approaches without access to the digital therapeutic intervention. After 12 weeks, control group participants received identical intervention access.

### Outcomes

The primary outcome was change from baseline in the International Consultation on Incontinence Questionnaire-Short Form (ICIQ-UI-SF) score, assessed at weeks 12, 24, 36, and 48. The ICIQ-UI-SF is a validated, widely-used patient-reported outcome measure for urinary incontinence symptom severity, consisting of three scored items assessing frequency of urinary leakage, amount of leakage, and overall impact on daily life, plus one unscored self-diagnostic item. Scores range from 0 to 21, with higher scores indicating greater symptom severity.^13^ Based on literature demonstrating that minimal clinically important differences (MCID) range from 1.5 points in app-based interventions to 2.5 points in conventional conservative therapies,^14^, ^15^ a reduction of ≥2 points was considered as clinically meaningful response. For descriptive analyses of symptom severity distribution, ICIQ-UI-SF total scores were categorised using a condensed three-level adaptation of the Klovning et al. categorisation as slight (0–5), moderate (6–12), or severe (13–21).^16^

Secondary outcomes included: (1) change from baseline in Incontinence Quality of Life (I-QoL) score, a validated 22-item disease-specific quality of life instrument with scores ranging from 0 to 100, where higher scores indicate better quality of life (MCID: 6.3 points)^17^; (2) continued independent pelvic floor exercise practice without app support; (3) initiation of new continence-related medications or treatments; and (4) objective app usage patterns from backend data. Objective backend engagement was captured as active minutes of app usage per content category per week.

Further exploratory outcomes included usage-response relationships between app engagement and clinical outcomes, examined through correlation analyses, regression modelling, and responder analyses based on objective usage metrics.

### Statistical analysis

The sample size was determined for the primary 12-week DINKS trial and is reported in full in the statistical analysis plan and primary publication.^9^ No separate calculation was performed for the present follow-up, which analyses the same fixed randomised sample (n = 194); all follow-up analyses are therefore exploratory rather than confirmatory.

The primary analysis compared groups at week 12 using analysis of covariance (ANCOVA) adjusting for baseline score. In the original trial, the intervention group showed a clinically meaningful reduction in ICIQ-UI-SF scores after 12 weeks compared with control, with a least squares mean difference of 3.7 points (95% CI 3.0–4.5; p < 0.0001), exceeding the minimal important difference.^9^ Given the waitlist design, we conducted time-aligned secondary analyses comparing outcomes at equivalent post-intervention intervals (e.g., intervention group at week 12 versus control group at week 24, both representing 12 weeks after intervention start). No non-inferiority or equivalence margin was prespecified; time-aligned comparisons are therefore reported as estimation only, without inference of equivalence.

Changes from baseline were analysed using ANCOVA. The baseline score was included as a covariate and treatment group as a factor. Adjusted mean differences, 95% confidence intervals, and p-values were calculated using estimated marginal means. Within-group changes were assessed using one-sample t-tests. Responder analyses were calculated with Clopper–Pearson exact 95% confidence intervals, and between-group differences were assessed using Fisher’s exact test.

The scores were analysed as (pseudo-)metric variables. Given their limited range and the relatively large sample sizes, the robustness of parametric procedures is approximately assured. Distributional assumptions were checked by graphical inspection of model residuals (Q–Q plots and histograms); medians (interquartile ranges) are reported alongside means.

The primary population followed the intention-to-treat (ITT) principle and comprised all 194 randomised participants (96 intervention, 98 control), analysed in their randomised groups with no post-randomisation exclusions. The safety population comprised all participants who accessed the app; no adverse events were reported. Because both groups ultimately received the identical intervention, no separate per-protocol efficacy population was defined. Intercurrent events were handled under a treatment-policy strategy: participants were retained in their randomised group with observed data unadjusted, and none were excluded or censored. The primary analysis used multiple imputation (MI) under a missing-at-random (MAR) assumption, implemented with the rbmi package (version 1.6.0) in R. The imputation model was a mixed-model for repeated measures (MMRM) fitted to observed data. Under MAR, 100 datasets were drawn from the posterior predictive distribution and analysed by ANCOVA; estimates were combined using Rubin’s rules. Complete-case (CC) analyses, defined as participants who provided data at the follow-up timepoints, were calculated and reported in the Supplementary materials including an additional MMRM analysis applied on the complete-cases set. As an additional sensitivity analysis, the MI-MAR was repeated with age category and incontinence subtype included as covariates in both the imputation and analysis models.

We assessed whether participants classified as nonresponders after the intervention were more likely to drop out at subsequent timepoints. Logistic regression models were performed separately for each group and follow-up timepoint, with dropout as the dependent variable and nonresponder status as the independent variable. Odds ratios (OR) and 95% confidence intervals (CI) were calculated. To further assess whether attrition was informative, we examined associations between loss to follow-up and baseline and early on-treatment characteristics (incontinence subtype, age, baseline ICIQ-UI-SF and I-QoL, early change from baseline in both outcomes, app satisfaction, comorbidity count, smoking, education, and prior physiotherapy) using univariable logistic regression at weeks 24, 36, and 48.

Exploratory usage–response analyses examined relationships between objective app usage metrics and clinical outcomes using Spearman rank correlations, with linear and logistic regression as sensitivity analyses. These analyses were post-hoc, not pre-specified in the original protocol, and are intended for hypothesis generation.

The DINKS statistical analysis plan was prespecified for the randomised phase of the trial and covers the analyses through the primary 12-week endpoint. The follow-up assessment timepoints (weeks 24, 36, and 48) were prespecified in the study protocol; however, the statistical analyses applied to these follow-up data were not specified in the analysis plan and are therefore exploratory. Accordingly, all analyses in this extended follow-up are descriptive and hypothesis-generating, and reported p-values are local, without adjustment for multiple comparisons. Statistical significance was set at two-sided α = 0.05. Analyses were conducted using R version 4.3.1 (R Foundation for Statistical Computing).

### Role of the funding source

The funder (Kranus Health GmbH) was the sponsor of the trial, provided the app-based therapy, and employed four authors (KM, LW, JEK, CPP) who were involved in data interpretation, manuscript writing, and the decision to submit the manuscript.

## Results

### Participant flow and baseline characteristics

Of 194 randomised participants, data availability decreased progressively over follow-up, with completion declining progressively from 186 (95.9%) at week 12–66 (34.0%) at week 48 (Table 1). At calendar week 48, observed data were available for 27 of 96 intervention group and 39 of 98 control group participants; the 48-week post-intervention estimate therefore derives from the intervention group only. The primary reasons for loss to follow-up were non-response to follow-up questionnaires despite email reminders. No participants withdrew due to adverse events. A CONSORT flow diagram detailing participant progression through the trial is provided in Fig. 1. Median age at baseline was 50 years (interquartile range (IQR) 40–57) and remained stable across follow-up timepoints (range: 50–54 years). Age distribution showed only minor fluctuations over time, with participants aged 45–60 years consistently representing the largest subgroup (44.3% at baseline, 54.5% at week 48).

**Table 1:** Data availability and age composition of the study cohort across follow-up–complete cases.

Timepoint	Participants available, n (%)	Age, median (IQR), years	<45 years, n (%)	45–60 years, n (%)	>60 years, n (%)
Baseline	194/194 (100%)	50 (40–57)	76 (39.2%)	86 (44.3%)	32 (16.5%)
Week 12	186/194 (95.9%)	50 (40–58)	71 (38.2%)	83 (44.6%)	32 (17.2%)
Week 24	122/194 (62.9%)	53 (40–59)	44 (36.1%)	55 (45.1%)	23 (18.9%)
Week 36	112/194 (57.7%)	50 (40–58)	44 (39.3%)	50 (44.6%)	18 (16.1%)
Week 48	66/194 (34.0%)	54 (41–58)	20 (30.3%)	36 (54.5%)	10 (15.2%)

N–number of participants; IQR–interquartile range.

Percentages for age categories are calculated using the number of participants with available age data at each timepoint.

**Fig. 1:**
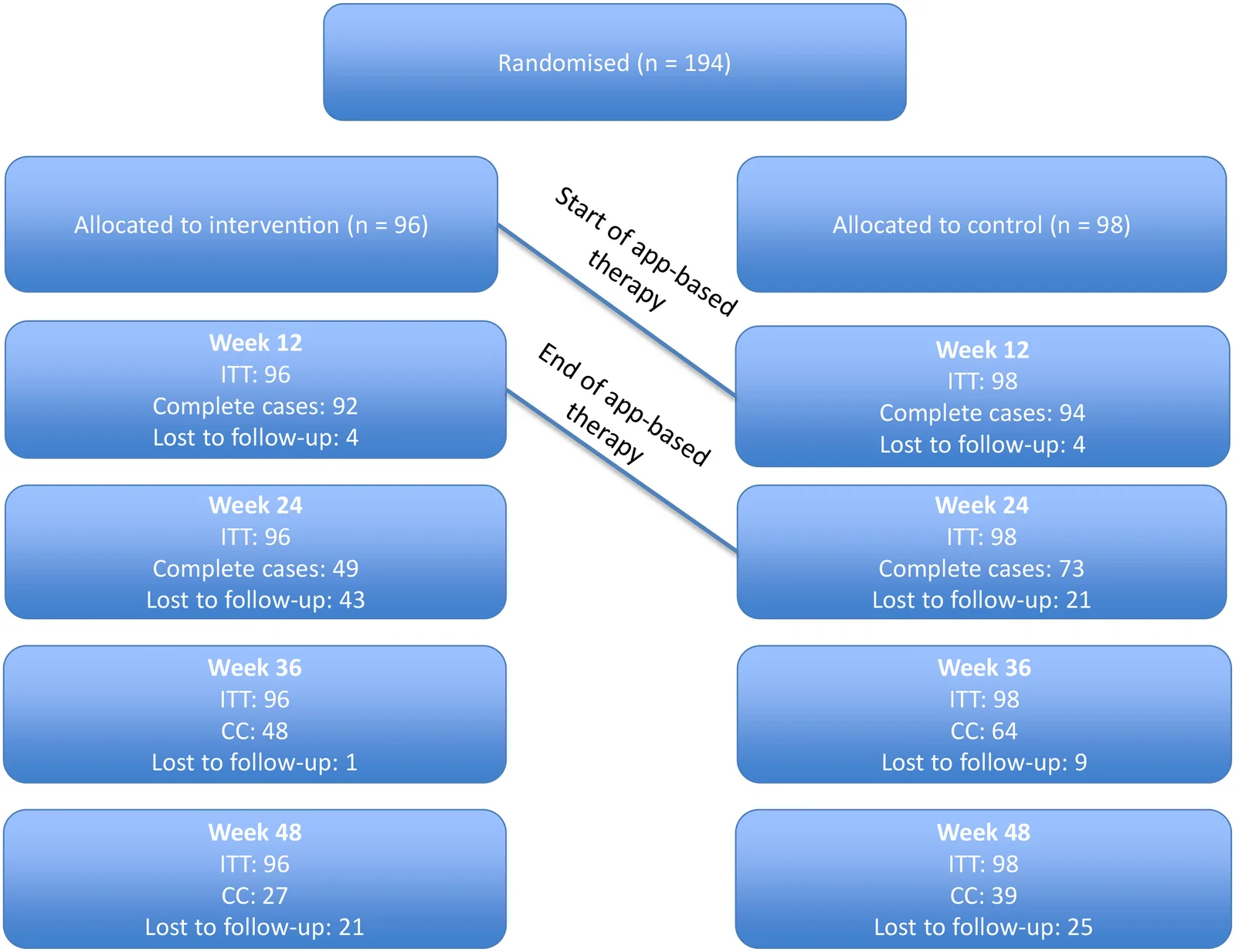
**CONSORT flow diagram.** Flow diagram showing participant progression through the trial from enrolment through 48-week follow-up. The intervention group (IG) received immediate app-based therapy; the control group (CG) received therapy after a 12-week waitlist period. ITT = intention-to-treat. CC = complete cases. N = number of participants. Full analysis set/ ITT (primary): all randomised participants (n = 194), analysed as randomised, missing data handled by MI-MAR. Complete-case set (sensitivity): participants with observed data at the relevant timepoint. Safety set: all participants who accessed the app; no adverse events were reported in either group, so a separate safety tabulation was not warranted.

Loss to follow-up was largely unrelated to participant characteristics. Neither baseline symptom severity (ICIQ-UI-SF; all p ≥ 0.34) nor quality of life (I-QoL; all p ≥ 0.17) predicted dropout at any timepoint, and measures of early treatment response were likewise unrelated (early ICIQ-UI-SF change all p ≥ 0.24; early I-QoL change all p ≥ 0.15). Nonresponder status was not consistently associated with dropout (intervention group odds ratio (OR) 1.00–1.69, all non-significant; Supplementary Tables A1–A3), with an association in the control group only at week 36 (OR 5.62, 95% CI 1.80–18.56, p = 0.003). Other associations were isolated to single timepoints: a weak age effect at week 24 (OR 0.97 per year, 95% CI 0.95–1.00; p = 0.021) and lower dropout among participants with stress compared with mixed incontinence at week 36 (OR 0.34, 95% CI 0.15–0.77; p = 0.010), the latter corresponding to a modest rise in the proportion of stress incontinence across follow-up (62.4% to 71.4%). Consistent with the week-24 age effect, completers and dropouts differed in age only at week 24 (median 53.0 versus 46.5 years; those lost were slightly younger; p = 0.018), with no difference at weeks 36 or 48 (Supplementary Table A4). None of these associations persisted across timepoints, indicating that attrition was not systematically related to any single characteristic. Overall, these analyses provide no evidence that attrition was driven by baseline disease burden or by poor early treatment response.

In the intervention group (IG; n = 96), both ICIQ-UI-SF and I-QoL showed clinically meaningful improvement after the therapy programme was finalised. For ICIQ-UI-SF, the mean change from intervention start was −4.09 points (95% CI −4.28 to −3.91) at week 12, and remained stable throughout follow-up (range −3.88 to −4.07; all p < 0.001; Table 2). For I-QoL, mean improvement was +18.85 points (95% CI + 17.92 to +19.78) at week 12, and similarly sustained (range +16.79 to +18.97; all p < 0.001; Table 2). These improvements exceeded predefined MCID thresholds at all timepoints, with the lower bound of the 95% confidence interval above the MCID.

**Table 2:** Change from baseline per group at follow-up.

Timepoint	n (mean)	Mean score (SE)	Mean CfB (SE)	95% CI	p-value
ICIQ-UI-SF					
Baseline–Late App (CG)	98	11.69 (0.29)	–	–	–
Baseline–Early App (IG)	96	11.51 (0.25)	–	–	–
12 wks–Late App (CG)	98	8.43 (0.39)	−3.23 (0.22)	−3.66, −2.80	<0.001
12 wks–Early App (IG)	96	7.42 (0.40)	−4.09 (0.09)	−4.28, −3.91	<0.001
24 wks–Late App (CG)	98	7.47 (0.38)	−4.17 (0.23)	−4.63, −3.71	<0.001
24 wks–Early App (IG)	96	7.65 (0.41)	−3.92 (0.29)	−4.48, −3.35	<0.001
36 wks–Late App (CG)	98	7.45 (0.49)	−4.20 (0.35)	−4.88, −3.52	<0.001
36 wks–Early App (IG)	96	7.46 (0.48)	−4.07 (0.34)	−4.74, −3.40	<0.001
48 wks–Early App (IG)	96	7.42 (0.51)	−3.88 (0.43)	−4.74, −3.03	<0.001
I-QoL					
Baseline–Late App (CG)	98	56.06 (2.47)	–	–	–
Baseline–Early App (IG)	96	58.35 (2.06)	–	–	–
12 wks–Late App (CG)	98	70.97 (2.48)	14.69 (1.01)	12.70, 16.67	<0.001
12 wks–Early App (IG)	96	77.17 (1.89)	18.85 (0.47)	17.92, 19.78	<0.001
24 wks–Late App (CG)	98	72.54 (2.30)	16.49 (1.03)	14.48, 18.51	<0.001
24 wks–Early App (IG)	96	76.67 (2.05)	18.57 (1.16)	16.29, 20.84	<0.001
36 wks–Late App (CG)	98	74.65 (2.47)	18.42 (1.79)	14.92, 21.93	<0.001
36 wks–Early App (IG)	96	77.25 (2.08)	18.97 (1.43)	16.17, 21.78	<0.001
48 wks–Early App (IG)	96	75.80 (2.91)	16.79 (2.86)	11.17, 22.41	<0.001

N-number of participants; SE–Standard Error; CfB–Change from Baseline; CI–confidence interval; IG–intervention group; CG–control group; wks–weeks; ICIQ-UI-SF–International Consultation on Incontinence Questionnaire–Urinary Incontinence Short Form; I-QoL–Incontinence Quality of Life.

MI-MAR imputed means per group. p-value: test CfB versus 0 (paired Rubin-pooled t-test). CfB relative to own intervention start (IG = W0, CG = W12). Week 48 is available for the IG only.

The control group (CG; n = 98) received the app-based therapy after a 12-week waitlist period. Following intervention start, the CG showed clinically meaningful and sustained improvements for both outcomes. For ICIQ-UI-SF, mean change from baseline ranged from −3.23 to −4.20 points across follow-up, and for I-QoL from +14.69 to +18.42 points (all p < 0.001; Table 2).

At baseline, ICIQ-UI-SF severity was comparable between groups, with most participants moderate (IG 62.5%; CG 58.2%) or severe (IG 37.5%; CG 41.8%) and none slight. After 12 weeks of app use, the IG shifted markedly toward milder symptoms (slight 27.2%; severe 12.0%), whereas the still-waiting CG was unchanged (slight 0%; severe 41.5%). A comparable shift emerged in the CG by week 24 (12 weeks after its app start; slight 11.0%; severe 16.4%), with both groups moving toward milder categories thereafter. Of note, the slight category (ICIQ-UI-SF 0–5) also includes participants who reported no incontinence symptoms (score 0); these were not differentiated from those with mild residual symptoms. Full distributions and per-visit sample sizes are provided in Supplementary Table A5 and Supplementary Fig. A1.

Because the control group initiated the intervention at week 12, we conducted time-aligned comparisons measuring outcomes at equivalent intervals after intervention start. At 12 weeks post-intervention, the primary MI-MAR analysis showed no significant between-group difference for ICIQ-UI-SF (adjusted mean difference −0.90, 95% CI −1.83 to 0.03; p = 0.059) but a significant difference in favor of the IG for I-QoL (adjusted mean difference +4.89 points, 95% CI + 0.34 to +9.44; p = 0.035; Table 3).

**Table 3:** Time-aligned between-group comparisons at equivalent post-intervention interval.

Outcome	Timepoint	n (IG)	n (CG)	Adj. mean diff. (IG−CG)	95% CI	p-value
ICIQ-UI-SF	Week 12	96	98	−0.90	−1.83, 0.03	0.059
	Week 24	96	98	0.18	−0.81, 1.17	0.719
	Week 36	96	98	0.08	−1.09, 1.25	0.892
I-QoL	Week 12	96	98	4.89	0.34, 9.44	0.035
	Week 24	96	98	2.91	−1.61, 7.44	0.207
	Week 36	96	98	1.50	−3.65, 6.64	0.568

N-number of participants; CI–confidence interval; IG–intervention group; CG–control group; ICIQ-UI-SF–International Consultation on Incontinence Questionnaire–Urinary Incontinence Short Form; I-QoL–Incontinence Quality of Life.

Time-aligned comparisons: IG weeks 12/24/36 versus CG weeks 24/36/48 (12/24/36 weeks post-intervention start). ANCOVA adjusted for baseline score and treatment group. MI-MAR = 100 imputations (rbmi), Rubin’s rules. Full randomised sample (n = 96 IG, n = 98 CG).

The I-QoL difference attenuated and was no longer significant at week 24 or week 36, while ICIQ-UI-SF remained non-significant at both timepoints (Table 3). These estimates were smaller than the minimal clinically important difference; for ICIQ-UI-SF the confidence intervals also excluded differences of that size, whereas for I-QoL the upper bounds did not. Within-group improvements were sustained across follow-up (Fig. 2). Supplementary sensitivity analyses using complete-case ANCOVA and mixed-model repeated measures (MMRM) without imputation, and a MI-MAR analysis with age and incontinence subtype included in the imputation model yielded consistent results (Supplementary Table A6 and Supplementary Fig. A2). Results pooled across both groups are shown in Table 4 and Fig. 3. Age structure did not differ significantly across incontinence subtypes (Fisher’s exact p = 0.26; Supplementary Table A7), and the two stratification factors were therefore largely independent, supporting their simultaneous adjustment in the sensitivity analysis.

**Fig. 2:**
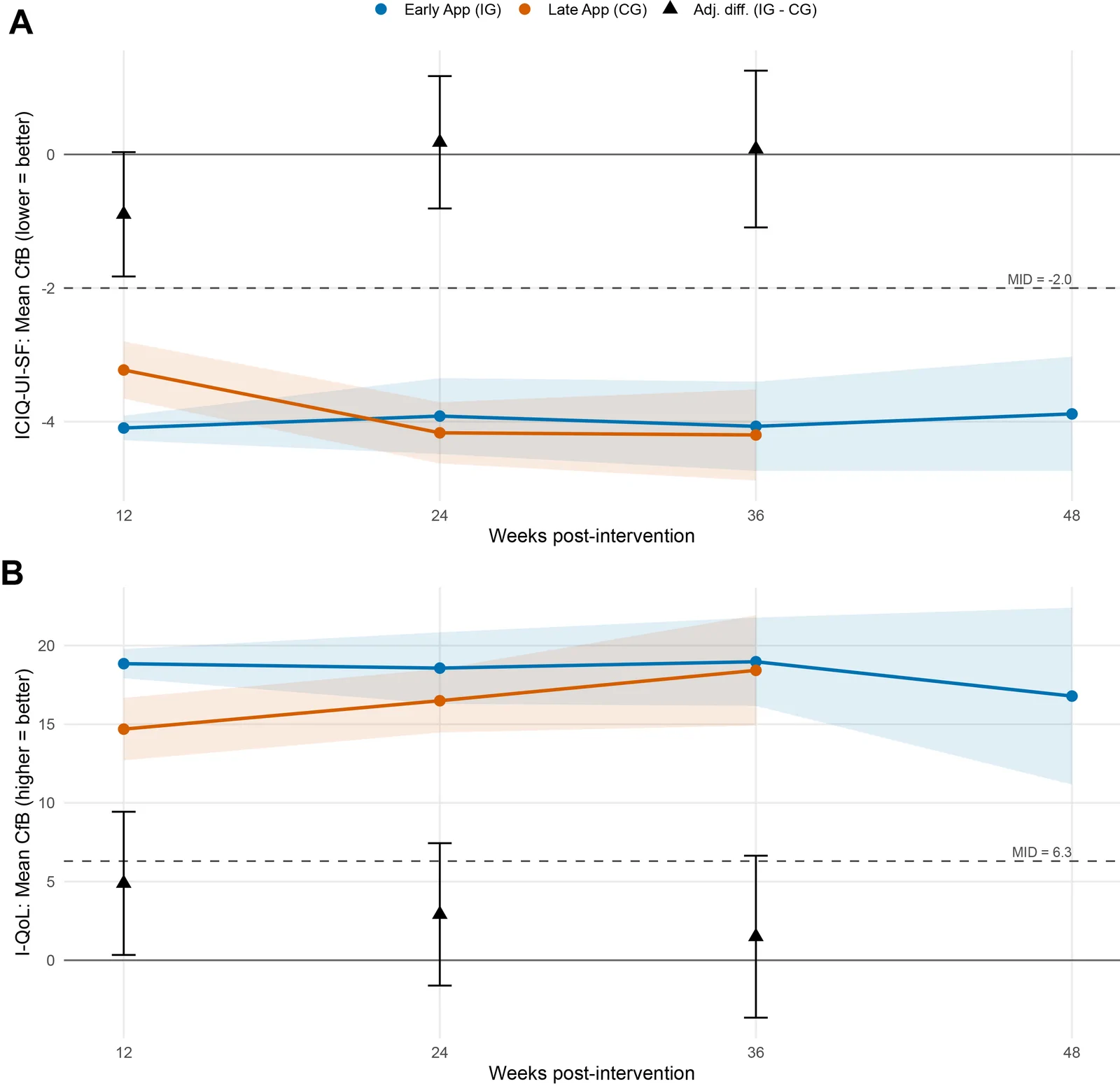
**Time-aligned between-group comparisons of change from baseline over 48 weeks.** CfB–Change from Baseline; CI–confidence interval; IG–intervention group; CG–control group; ICIQ-UI-SF–International Consultation on Incontinence Questionnaire–Urinary Incontinence Short Form; I-QoL–Incontinence Quality of Life. (A) ICIQ-UI-SF scores. (B) I-QoL scores. Data are MI-MAR imputed means with shaded areas representing 95% confidence intervals. The intervention group (blue) is plotted by calendar week; the control group (orange) is time-aligned to intervention start (i.e., control group week 24 is aligned with intervention group week 12, both representing 12 weeks post-intervention). The black horizontal line indicates null (no change); the dashed red line indicates the minimal clinically important difference (ICIQ-UI-SF: 2.0 points; I-QoL: 6.3 points). Black triangles indicate MI-MAR–adjusted mean differences between groups at corresponding post-intervention timepoints. Incontinence Short Form. I-QoL = Incontinence Quality of Life questionnaire. MI-MAR = multiple imputation under missing-at-random assumption.

**Fig. 3:**
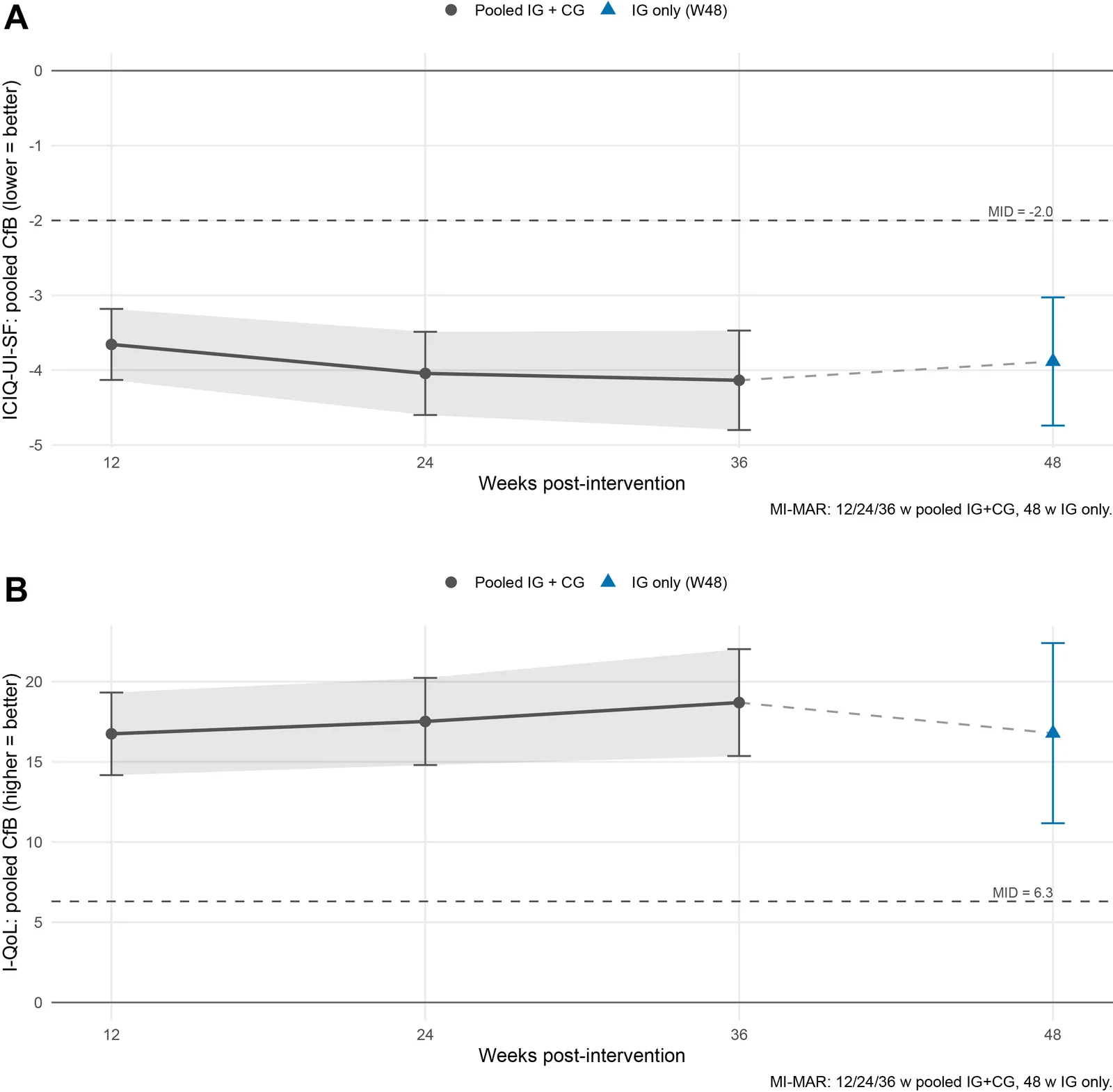
**Pooled cohort trajectory of change from baseline aligned to intervention start.** CfB–Change from Baseline; CI–confidence interval; IG–intervention group; CG–control group; ICIQ-UI-SF–International Consultation on Incontinence Questionnaire–Urinary Incontinence Short Form; I-QoL–Incontinence Quality of Life; w-week. (A) ICIQ-UI-SF scores. (B) I-QoL scores. Both groups are combined and aligned to their respective intervention start dates (intervention group: week 0; control group: week 12). Data are MI-MAR imputed means (n = 194 for 12–36 weeks post-intervention; n = 96 [IG only] for 48 weeks post-intervention, 27 of 96 with observed data) with shaded areas representing 95% confidence intervals. The black horizontal line indicates null (no change); the dashed line indicates the minimal clinically important difference. All improvements exceeded the MCID at every timepoint (all p < 0.001).

**Table 4:** Combined cohort change from baseline — time-aligned (primary MI-MAR analysis).

Outcome	Post-intervention interval	n	Mean CfB (95% CI)	p-value
ICIQ-UI-SF	12 wks post-int.	194	−3.66 (−4.13, −3.18)	<0.001
	24 wks post-int.	194	−4.04 (−4.60, −3.49)	<0.001
	36 wks post-int.	194	−4.13 (−4.80, −3.47)	<0.001
	48 wks post-int.^a^	96	−3.88 (−4.74, −3.03)	<0.001
I-QoL	12 wks post-int.	194	+16.75 (+14.17, +19.32)	<0.001
	24 wks post-int.	194	+17.52 (+14.80, +20.24)	<0.001
	36 wks post-int.	194	+18.70 (+15.36, +22.03)	<0.001
	48 wks post-int.^a^	96	+16.79 (+11.17, +22.41)	<0.001

CfB–Change from Baseline; CI–confidence interval; ICIQ-UI-SF–International Consultation on Incontinence Questionnaire–Urinary Incontinence Short Form; I-QoL–Incontinence Quality of Life; wks–weeks.

Combined cohort (IG + CG pooled, time-aligned to intervention start) for 12–36 weeks post-intervention. CfB = change from baseline. MI-MAR = 100 imputations (rbmi), full randomised sample (n = 194). p-value tests H0: mean CfB = 0.

a 48-week data: IG only (no CG equivalent) and 27 of 96 with observed data; imputed under MAR via 4-visit MMRM (Rubin’s rules).

### Responder analysis

Responder rates were high and stable across follow-up for both outcomes in both groups (Table 5). Using the primary MI-MAR analysis, ICIQ-UI-SF responder rates in the IG ranged from 73.3% to 77.3% across post-intervention timepoints (12–48 weeks; no control-group comparator exists at 48 weeks), while CG responder rates ranged from 76.1% to 77.0% (12–36 weeks). For I-QoL, IG responder rates ranged from 71.9% to 78.5%, and CG rates from 72.1% to 73.3% (Fig. 4).

**Table 5:** Responder rates — time-aligned post-intervention comparisons (primary MI-MAR analysis).

Outcome	Post-intervention interval	n (IG)	Responders IG % (95% CI)	n (CG)	Responders CG % (95% CI)	p-value
ICIQ-UI-SF	12 wks post-int.	96	74.9% (64.9–82.8)	98	76.1% (64.8–84.7)	0.851
	24 wks post-int.	96	73.3% (61.2–82.6)	98	76.6% (65.5–85.0)	0.642
	36 wks post-int.	96	73.8% (61.7–83.2)	98	77.0% (64.9–85.9)	0.678
	48 wks post-int.	96	77.3% (63.2–91.4)	–	–	–
I-QoL	12 wks post-int.	96	78.5% (68.9–85.8)	98	72.1% (61.3–80.9)	0.328
	24 wks post-int.	96	76.7% (65.4–85.1)	98	73.3% (62.2–82.1)	0.636
	36 wks post-int.	96	76.1% (65.0–84.5)	98	72.1% (60.1–81.7)	0.591
	48 wks post-int.	96	71.9% (57.6–86.3)	–	–	–

CfB–Change from Baseline; CI–confidence interval; IG–intervention group; CG–control group; ICIQ-UI-SF–International Consultation on Incontinence Questionnaire–Urinary Incontinence Short Form; I-QoL–Incontinence Quality of Life; wks–weeks.

Responder thresholds: ICIQ-UI-SF ≥2.0-point reduction from baseline; I-QoL ≥ 6.3-point increase from baseline. MI-MAR proportions pooled via logit transformation (Rubin’s rules). p-value from pooled chi-square test. Time-aligned comparisons: IG weeks 12/24/36 versus CG weeks 24/36/48. Full randomised sample (n = 96 IG, n = 98 CG).

**Fig. 4:**
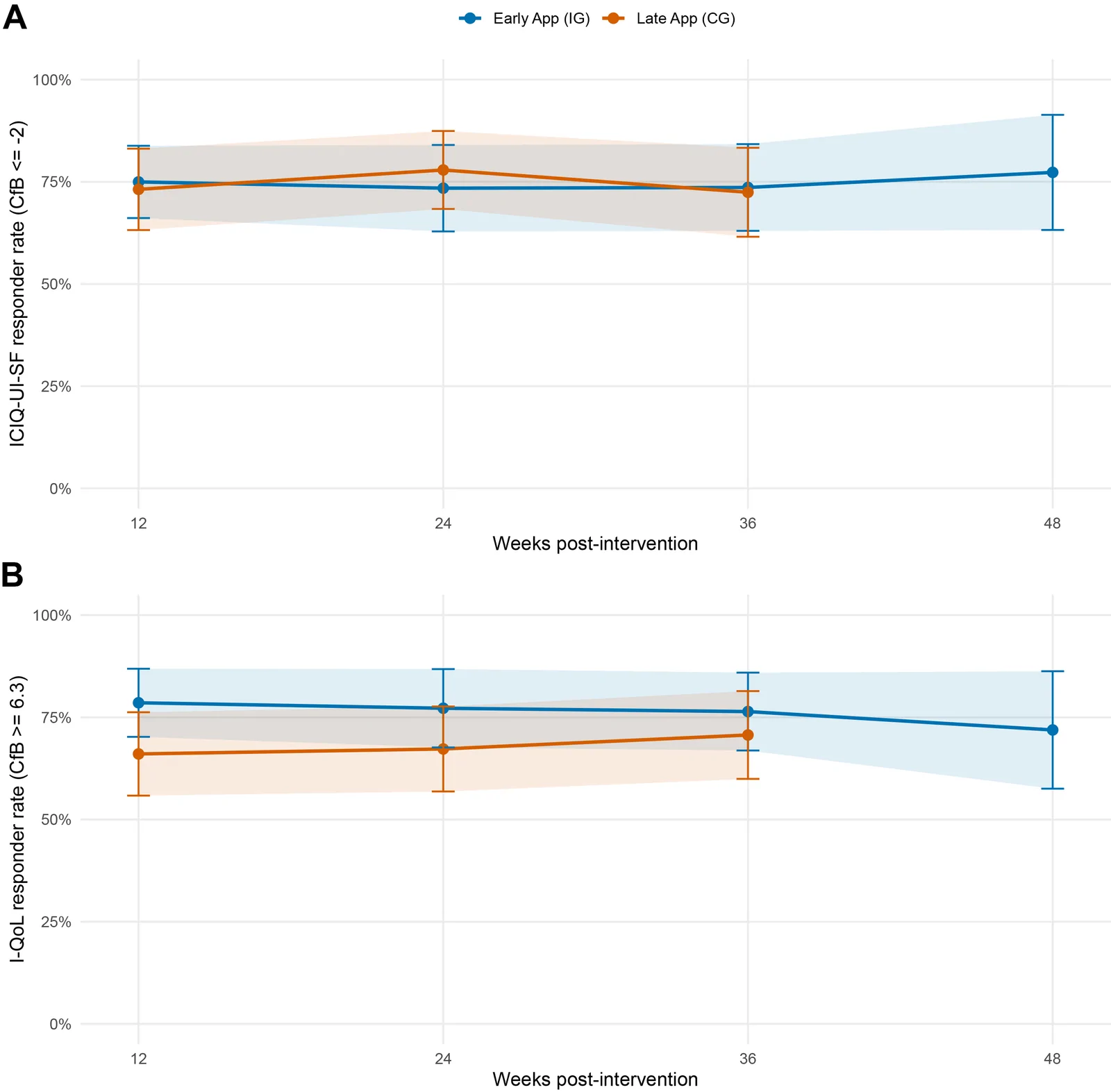
**Time-aligned responder rates at equivalent post-intervention timepoints.** CfB–Change from Baseline; ICIQ-UI-SF–International Consultation on Incontinence Questionnaire–Urinary Incontinence Short Form; I-QoL–Incontinence Quality of Life; MI-MAR = multiple imputation under missing-at-random assumption.(A) ICIQ-UI-SF responders (≥2.0-point reduction from baseline). (B) I-QoL responders (≥6.3-point increase from baseline). Data are MI-MAR pooled responder proportions with 95% confidence intervals (Clopper–Pearson) for the intervention group (blue) and control group (orange) at equivalent post-intervention intervals (12, 24, and 36 weeks after intervention start). The 48-week timepoint is available for the intervention group only. No statistically significant between-group differences were observed at any timepoint (all p > 0.3, pooled chi-square test).

No statistically significant between-group differences in responder rates were observed at any time-aligned post-intervention timepoint for ICIQ-UI-SF (all p > 0.6) or I-QoL (all p > 0.3; Table 5). Complete-case responder rates (Supplementary Table A9) were broadly consistent with MI-MAR estimates.

### Continuation of independent exercise practice and new treatments

At week 36, 65.8% (73/111) of participants with available data reported continuing pelvic floor exercises independently without app support. At week 48, 64.6% (42/65; one participant with available outcome data did not answer this item at each visit) continued independent practice, suggesting successful transition to self-management for approximately two-thirds of participants with available data at these visits.

Regarding initiation of new continence-related treatments during extended follow-up, at week 36, one participant initiated duloxetine, one initiated pelvic floor electrical muscle stimulation, and four reported using herbal preparations. At week 48, one participant reported duloxetine use, with no other new therapies initiated. Overall, rates of new therapy initiation remained low, supporting durability of app-based treatment effects not driven by additional continence-related treatments.

### Usage-response analyses

Across 179 participants with backend data, app usage was not correlated with ICIQ-UI-SF change at any timepoint (all ρ < 0.13, p > 0.16), category-specific usage showed no meaningful correlations, and early engagement was similar between responders and nonresponders (p > 0.30); sensitivity analyses were consistent (Supplementary Fig. A3). These findings may indicate a threshold effect rather than a usage-dependent response, whereby efficacy derives from behaviour-change initiation and content exposure rather than practice intensity.

## Discussion

This 48-week follow-up of the DINKS randomised controlled trial shows that app-based multimodal therapy for urinary incontinence was associated with substantial improvements that were sustained throughout follow-up, most reliably through 36 weeks after intervention start, with 72–79% of participants maintaining a clinically meaningful response. In time-aligned comparisons, estimated differences between immediate and 12-week-delayed initiation were small relative to the MCID. Because no margin was prespecified and the trial was not powered for such a comparison, these estimates describe what was observed and cannot establish that delayed initiation preserves effectiveness. Sustained benefit was observed after a single 12-week therapy cycle, with approximately two-thirds of those still providing data successfully transitioning to independent self-management.

The magnitude and durability of improvement with ICIQ-UI-SF reductions of 3.7–4.1 points sustained through 36 weeks aligns with previous long-term digital intervention studies reporting sustained improvements of 2.2–4.0 points at 4–24 months.^11^, ^18^, ^19^, ^20^ Our consistently high responder rates (72–79% across all timepoints) exceed those reported in some studies (56% at 3 months),^19^ likely reflecting our comprehensive multimodal approach integrating physical, behavioural, and psychological components, and potentially the rigorous regulatory authority approval standards enhancing treatment fidelity and patient trust.

These results suggest that digital therapeutics may help avoid or delay surgical intervention in some patients. This is consistent with meta-analytic evidence indicating that PFMT reduces the risk of therapy escalation, as well as studies showing that structured PFMT can postpone the need for surgery.^21^, ^22^, ^23^, ^24^ Surgical treatments (mid-urethral slings) achieve larger ICIQ-UI-SF improvements (8–10 points)^25^, ^26^ but carry procedural risks and potential complications. The consistency of our findings with previous digital intervention studies (Sjöström 3.6 points at 2 years,^20^ Hoffman 3.1 points at 2 years,^18^ Wadensten 4.0 points at 15 months,^11^ Loohuis 2.16 points at 4 months^19^) supports clinically meaningful and consistent improvements. While not replacing surgery when indicated, these approaches may offer meaningful symptom control with important health-economic implications.

Outcomes in the two groups were similar once both received app-based therapy (ICIQ-UI-SF at 36 weeks post-intervention: 0.08, 95% CI −1.09 to 1.25; p = 0.892), consistent with the treatment effect observed in the DINKS RCT before crossover,^9^ and no previous digital intervention study has examined delayed versus immediate access. European policy documents identify long waiting times as a critical barrier to accessing continence care, with calls for integrated community-based services.^2^ Our data, however, address only a single, fixed 12-week delay. Longer delays could plausibly have different consequences through symptom progression, declining motivation and engagement, or changes in care-seeking behaviour, which we cannot address. During the waiting period the control group’s severity distribution was essentially unchanged (slight 0%, severe 41.5%). A short, defined delay did not reduce the benefit eventually achieved in this cohort; this does not indicate that waiting is acceptable, and waiting times should be minimised wherever possible.

Our findings address broader women’s health equity priorities. Digital health technologies improve women’s access to care, self-monitoring, and health education, contributing to empowerment through skills acquisition.^27^ Our intervention exemplifies this: 65–66% of participants with available data successfully transitioned to independent self-management at the last two timepoints, demonstrating acquisition of skills and self-efficacy extending beyond the digital tool itself. However, our smartphone requirement underscores the importance of addressing digital literacy and connectivity gaps to ensure equitable access. Nevertheless, current population-level data suggest that such barriers may be diminishing: in the EU, 94% of individuals reported using the internet within the last three months in 2025, and almost nine in ten accessed it via a mobile device.^28^

The sustained improvements after therapy cessation suggest that comprehensive digital therapeutics produce lasting behavioural and physiological changes. Possible mechanisms include: learned motor patterns for pelvic floor recruitment becoming automatised; internalised cognitive behavioural strategies for urge control and anxiety management; sustained lifestyle modifications (fluid management, bladder training); and increased self-efficacy and health activation, as evidenced by PAM-13 improvements in the primary trial^9^ and the finding that 65–66% of participants with available data continued independent exercises without app support at late follow-up. The integration of educational content, behavioural strategies, and physiotherapy likely creates synergistic effects that persist after formal intervention completion.

Our exploratory usage-response analyses revealed no significant associations between app usage intensity and clinical outcomes, suggesting a possible threshold-based rather than usage-dependent effectiveness. This pattern, characteristic of cognitive behavioural therapy-based digital therapeutics, has important implications for patient counselling: clinical benefit does not require intensive daily engagement, potentially reducing barriers to adoption and facilitating equitable access across varying levels of digital literacy. Future studies with prospective usage-response designs are needed to confirm these exploratory findings.

### Several study limitations warrant consideration

First, attrition rates at later timepoints introduce potential selection bias, although our rates reflect a common challenge in long-term follow-up studies.^29^ Our primary analysis used multiple imputation under a missing-at-random assumption, with complete-case and mixed-model repeated-measures analyses giving consistent estimates. No association was detected between loss to follow-up and baseline symptom severity, quality of life, or early treatment response at any timepoint, and nonresponder status did not consistently predict dropout, the mechanisms most likely to bias durability estimates. The only associations detected were isolated to single timepoints (a weak age effect at week 24 and lower dropout among stress- versus mixed-incontinence participants at week 36), none persisting across follow-up, indicating that attrition was not systematically related to any single characteristic. The week-36 subtype association corresponded to a modest shift in subtype composition. In a sensitivity analysis including incontinence subtype and age category in both the imputation and analysis models, the treatment estimates were essentially unchanged, indicating that this did not materially affect the findings. Together with the stability of responder rates among participants with available data (73–77% for ICIQ-UI-SF and 72–79% for I-QoL across timepoints) and of baseline characteristics over follow-up, these observations are consistent with the missing-at-random assumption underlying our primary analysis. Nonetheless, these analyses cannot remove the uncertainty introduced by attrition, and informative (missing-not-at-random) dropout cannot be excluded. Precision also decreases progressively across follow-up: at calendar week 48 only 66 of 194 participants provided data, so the 48-week estimate is single-arm and hypothesis-generating rather than confirmatory. As participants received only email reminders, future studies should consider more intensive retention strategies to minimise attrition.

Second, the absence of an active comparator group limits conclusions about relative effectiveness versus in-person physiotherapy. However, the consistency of improvements across our study (3.7–4.1 points) and previous digital interventions (Sjöström 3.6–3.7 points,^20^ Hoffman 3.1 points,^18^ Wadensten 4.0 points,^11^ Loohuis 2.16 points^19^) suggests comparable outcomes with traditional therapy, and Loohuis and colleagues’ demonstration of non-inferiority to usual care^19^ provides supporting evidence. Future trials should directly compare app-based interventions with gold-standard supervised physiotherapy to establish non-inferiority or equivalence.

Third, objective measures such as pad weights were not collected at follow-up, though validated patient-reported outcomes (ICIQ-UI-SF, I-QOL) serve as accepted endpoints in incontinence research, with evidence suggesting that the ICIQ-UI-SF could be an appropriate substitute for the 1-h pad weight testing in predicting the severity of urinary incontinence.^30^ The pad usage reduction demonstrated in the primary trial (2.6 pads over 3 days, p < 0.0001)^9^ and Hoffman’s finding of significant reductions in incontinence protection product use at 2-year follow-up (p = 0.04)^18^ support the validity of sustained patient-reported improvements.

Fourth, our cohort was recruited through specialist urology and gynaecology offices across Germany, was predominantly born in Germany (88%),^9^ and required smartphone or tablet ownership; findings may therefore not generalise to primary-care, treatment-naïve, or ethnically diverse populations. Race and ethnicity, sociocultural constructs rather than biological traits, were not recorded; any differences across populations would more likely reflect unmeasured structural and socioeconomic factors (digital access and literacy, language, care-seeking behaviour) than ancestry. As a scalable, self-directed intervention, app-based therapy nonetheless has potential to widen access for underserved groups, provided digital-access gaps are addressed. Future studies should validate findings in more diverse populations, particularly regarding potential differences in digital literacy, treatment preferences, and care-seeking behaviours across ethnic and cultural groups, which is essential for addressing the digital gender gap and ensuring equitable access to digital health technologies.^27^

Fifth, the exploratory usage-response analyses were not prespecified in detail and should be interpreted as hypothesis-generating rather than confirmatory. Prospective studies specifically designed to examine usage-response relationships are needed to confirm these patterns.

Despite these limitations, our study together with the growing evidence base from previous research^11^, ^18^, ^19^, ^31^ supports the durability of app-based therapy for urinary incontinence. Our findings suggest threshold-based rather than usage-dependent effectiveness, and feasibility of transition to independent self-management, collectively addressing critical evidence gaps identified in international policy documents calling for cost-effective, sustainable continence care solutions.^2^ Whether digital therapeutics can reduce progression to surgical treatment remains an important question that future prospective studies with surgical referral as a prespecified endpoint should address.

Future research should prioritise: (1) identification of predictors determining individual therapy duration requirements; (2) component-specific analyses to determine which intervention elements contribute most to sustained improvement; (3) cost-effectiveness analyses incorporating long-term benefit maintenance, reduced pad usage, and avoided surgical progression; (4) examination of optimal strategies for non-responders; (5) prospective comparison of different delay durations before initiation; (6) assessment of whether digital therapeutics reduce progression to invasive treatments; and (7) validation in diverse international populations to ensure equitable access.

In conclusion, this 48-week follow-up shows that app-based multimodal therapy can produce substantial, clinically meaningful improvements in urinary incontinence that were maintained through at least 36 weeks after intervention start and were consistent across sensitivity analyses, with no detected association between loss to follow-up and baseline severity, quality of life, or early treatment response. As the week-48 assessment was available for the intervention group only, and for 27 of 96 participants, this timepoint is exploratory and hypothesis-generating. Estimated differences between immediate and 12-week-delayed initiation were small relative to the MCID, but no non-inferiority margin was prespecified, only one delay duration was evaluated, and symptoms were unchanged while waiting; waiting times should therefore still be minimised. Sustained benefit followed a single 12-week cycle, with approximately two-thirds of those still providing data transitioning to independent self-management, and exploratory analyses suggested a possible threshold rather than usage-dependent effect, warranting prospective confirmation.

Together, these results support digital therapeutics as a promising conservative treatment option for female urinary incontinence across all subtypes, addressing access barriers through privacy and scalability. Larger trials with active comparators, longer follow-up, and prespecified analyses of surgical progression are needed to confirm and extend these findings.

## Contributors

LF, AH, KM, LW, LR, and CPP conceived and designed the follow-up study. LF wrote the original draft of the manuscript. LF and AH performed quality assessment and data interpretation. WL and CPP designed the statistical analysis plan and performed statistical analyses. LF, GD, and JHB conducted investigations and managed data collection. KAB, LR, and DM managed patient recruitment and screening. CPP, LW, KM, and JEK contributed to data interpretation and project administration. WL, LF, GD, CPP, LW, and AH directly accessed and verified the underlying data reported in the manuscript. All authors critically reviewed and revised the manuscript and approved the final version to be published. All authors had full access to all the data in the study.

## Data sharing statement

De-identified individual participant data will be made available upon request to researchers who provide a methodologically sound proposal, beginning 6 months after publication, with no end date. Study protocols and statistical analysis plans will also be available. Proposals should be directed to studie@kranus.de to gain access and data requestors will need to sign a data access agreement.

## Declaration of interests

CPP, KM, JEK, and LW are employed by Kranus Health. WL provides statistical counselling for Kranus Health. AH, LF, GD and JHB institution received funding from Kranus Health to conduct the study. LF received honoraria from Bayer Vital for presentation and support for attending meetings from Kranus Health. GD has received a grant funding from the German Federal Ministry of Education and Research and the Else-Kröner-Fresenius-Stiftung. GD received honoraria from Johnson&Johnson Corporation, Merck KGaA, and Boston Scientific Corporation for presentations and lectures. DEM received consulting fees and honoraria for presentation from Kranus Health. KAB received consulting fees and honoraria for presentation from Kranus Health, BTL and Gedeon Richter. All other authors declare no competing interests.

## Appendix A Supplementary data

Studyprotocol2.1_DINKS_signed

SAP_final_DINKS

Supplementary Figs. A1–A3 and Tables A1–A9
