# Supplementary material for: Long-term effectiveness of app-based therapy for female urinary incontinence: 48-week follow-up of a randomised controlled trial
Source: eClinicalMedicine. 2026 Oct 28;101:104298. doi: 10.1016/j.eclinm.2026.104298 (PMC13735171; doi:10.1016/j.eclinm.2026.104298)
Supplement: Supplementary file 1 [file mmc1.pdf]

Docusign Envelope ID: 05A6509E-2636-42E1-8D3B-8B2D12CE3674

Study protocol

Prospective randomised study on multimodal self-treatment of women with incontinence complaints using a digital health application

DINKS (Digital Incontinence Study)

This document is a translated version of the original German protocol.
While every effort has been made to ensure accuracy,
minor language discrepancies may occur.

Study acronym: DINKS (Digital Incontinence Study) Protocol
version: 2.1 final
Confidentiality note: The content of this study protocol is confidential and may not be disclosed to unauthorised persons either verbally or in writing.

Table of contents

Table of contents **2**

Version history **5**

1 General information **6**

1.1 Persons, institutions and bodies involved *6*

1.2 Signatures *7*

1.3 Summary *8*

1.4 Study synopsis *9*

1.5 Business round scheme *16*

2 List of abbreviations **17**

3 Test population **19**

3.1 Inclusion criteria *19*

3.2 Exclusion criteria *19*

4 Question and background **21**

4.1 Forms of urinary incontinence *21*

4.1.1 Stress incontinence 21
4.1.2 Urge incontinence 21
4.1.3 Mixed incontinence 21

4.2 Distribution and frequency *21*

4.3 Diagnosis of urinary incontinence *22*

4.4 Treatment of urinary incontinence *23*

4.4.1 Underlying diseases and medication 23
4.4.2 Lifestyle changes 23
4.4.3 Physiotherapy/ pelvic floor training 23
4.4.4 Bladder training 24
4.4.5 Drug therapy 24
4.4.6 Digital therapy programmes 24

4.5 Conclusions and rationale for the study *25*

5 Study endpoints and supply effects **27**

5.1 Incontinence episodes frequency (IEF) *27*

5.2 Questionnaires *27*

5.2.1 ICIQ-SF Questionnaire 27
5.2.2 I-QOL questionnaire 28
5.2.3 PAM-13 Questionnaire 28
5.2.4 PGI-I questionnaire 28
5.2.5 S/U-IQ questionnaire 28
5.2.6 Further questions about app usage 29

5.3 Therapy failure *29*

6 Study design and description 30

6.1 Type of study, therapy allocation, scope of the study 30

6.2 Patient recruitment 30

6.3 Timetable 30

6.4 Study centre 30

6.5 Study programme 30

6.6 Patient insurance 31

6.7 Screening, information and consent 31

6.8 Pre-screening, medical history, inclusion procedure 32

6.9 Treatment phase 32

6.9.1 Micturition diary 33

6.9.2 Pelvic floor and physiotherapy exercises 33

6.9.3 Bladder training / cognitive behavioural therapy 33

6.9.4 Mental exercises 33

6.9.5 Acute urge control 33

6.9.6 Knowledge section /Nutrition section 34

6.9.7 Further functions: 34

6.10 Visits and analyses 34

6.11 End of study participation 34

6.12 End of the study 34

7 Adverse events 35

7.1 Complications and risks 35

7.2 Recording and documentation of adverse events 35

7.3 Adverse event 35

7.4 Serious Adverse Event 35

7.5 Product defect or device-related adverse events 35

7.6 Definition of the risk threshold 36

7.6.1 Risk threshold for patients 36

7.6.2 Risk threshold for the study 36

7.6.3 Occurrence of a pregnancy 36

8 Statistics 37

8.1 End points 37

8.2 Primary endpoint 37

8.3 Secondary endpoints 37

8.4 Planning the scope of the study (case number planning) 37

8.5 Discussion of the endpoints 38

8.6 Primary endpoint 38

8.7 Secondary endpoints 38

8.8 Statistical analysis 39

Docusign Envelope ID: 05A6509E-2636-42E1-8D3B-8B2D12CE3674

8.9 Presentation of the results *41*

8.10 Tables *41*

8.11 Listings *41*

8.12 Data collection/documentation forms *42*

9 Data management 43

9.1 Patient identification *43*

9.2 Data acquisition *43*

9.3 Data protection *43*

9.4 Quality assurance *43*

9.5 Archiving *44*

10 Ethical issues, legal and administrative regulations 45

10.1 Declaration of Helsinki and Good Clinical Practice *45*

10.2 Independent ethics committees *45*

10.3 Legal regulations *45*

10.4 Registration *45*

10.5 Financing *45*

10.6 Final report and publication *45*

11 Bibliography 46

A. Appendix 48

A1. Questionnaires used *48*

A1.1 PGI-I: Patient Global Impressions Scale - Improvement 48

A1.2 I-QOL: Urinary Incontinence Quality of Life Scale 49

A1.3 ICIQ-SF: International Consultation on Incontinence Questionnaire-Urinary Incontinence Short Form 53

A1.4 PAM-13: Patient Activation Measure 54

A1.5 S/U-IQ: Stress/Urge Incontinence Questionnaire and Classification System 56

Docusign Envelope ID: 05A6509E-2636-42E1-8D3B-8B2D12CE3674

Version history

Version	Section	Changes	date
1.0	-	Initial version	05.03.2024
2.0	Entire document	Editorial adjustments	22.07.2024
	1.4 Study synopsis<br>3.2 Exclusion criteria	Specification of the exclusion criterion "OP small pelvis"	
	1.4 Study synopsis<br>6.3 Schedule<br>10.4 Final report and publication	Extension of the recruitment period, corresponding adjustment of the schedule	
2.1	1.4 Study synopsis<br>3.2 Exclusion criteria<br>8.3 Secondary endpoints	Two additional secondary endpoints were added, control group was described in more detail	14.11.2024
	5.3 Therapy failure	Definition of treatment failure was specified in more detail	
	8.8 Statistical analysis	Subgroup analyses were carried out in more detail<br>specified	
	A1.2 Appendix	I-QOL: Urinary Incontinence Quality of Life Scale has been corrected and completed	

1 General information

1.1 Persons, institutions and bodies involved

**Scientific management:**<br>Prof Dr med Axel Haferkamp<br>Urological University Clinic Mainz	**Statistics:**<br>Prof. Dr rer. nat. Walter Lehmacher<br>Self-employed statistician, former director<br>Institute for Med. Statistics, Informatics and<br>Epidemiology (IMSIE) at the University of Cologne<br>Robert-Koch-Str. 10 - Building 55<br>50937 Cologne<br><br>Dipl.-Stat. Michael Bulitta<br>CRM Biometrics GmbH<br>Mary-Anderson-Str. 6<br>53332 Bornheim
**Protocol Committee:**<br>Prof Dr Axel Haferkamp Prof Dr<br>Kurt Miller<br>Dr Laura Elisa Wiemer<br><br>**CRO:**<br>CW-Research & Management GmbH<br>Konrad-Zuse-Platz 8<br>81829 Munich<br><br>**Sponsor:**<br>Kranus Health GmbH<br>Westenriederstr. 10<br>80331 Munich	**Monitoring:**<br>n. a.<br><br>**Data management and project coordination:**<br>Heike Kuse<br><br>**Laboratory/s:**<br>n. a.
**Reference institutes:**<br>n. a.	

Docusign Envelope ID: 05A6509E-2636-42E1-8D3B-8B2D12CE3674

1.2 Signatures

DocuSigned by:
Prof. Dr. med. Axel Haferkamp 2024
2572F51310D14CD...

Prof. Dr Axel Haferkamp Scientific Director

DocuSigned by:
Prof. Dr. Walter Lehmacher 29.11.2024
D92954412652489...

Prof. Dr rer. nat. Walter Lehmacher
Statistician

DocuSigned by:
Dipl.Stat. Michael Bulitta 29.11.2024
A465CB0A788A4EF...

Dipl.-Stat. Michael Bulitta
Statistician

DocuSigned by:
28.11.2024
92B1BA59491045F...

Dr Laura Elisa Wiemer
On behalf of the sponsor, Kranus Health GmbH

Docusign Envelope ID: 05A6509E-2636-42E1-8D3B-8B2D12CE3674

1.3 Summary

Urinary incontinence in women is a very common clinical picture in which uncontrolled urine loss is the main symptom. Incontinence complaints are divided into stress incontinence, urge incontinence and mixed urinary incontinence. In Germany, around 15-25% of women are affected by various forms of urinary incontinence [1]. The frequency increases with age. A high number of unreported cases can be assumed [2]. Various treatment options are available for the different forms of incontinence, including lifestyle changes, physiotherapy and pelvic floor muscle training, micturition monitoring, as well as medication and finally surgical procedures.

Despite the recommendations of the guidelines [2, 3] and proven effectiveness [4] as a primary therapy, comprehensive, multimodal therapy and monitoring programmes (bladder diary, pelvic floor training, lifestyle changes, fluid management, bladder training) for female incontinence are rarely offered in clinical practice. The reason for this is the complexity of organisation and implementation in the "analogue" world. Although the attending physician has contact persons (e.g. physiotherapists) available for the individual modules, close and cross-module monitoring of patients, which is essential for the success of treatment [2, 5], cannot be realised in clinical practice. A digital health application (DiGA) can make it possible to implement the treatment concepts recommended in the guidelines and thus close a gap in care.

The positive care effect of such a DiGA is to be demonstrated in a randomised study for patients with stress incontinence (N39.3), urge incontinence (N39.42) and mixed forms of the two types of incontinence. In the intervention arm, patients receive access to a 12-week digital therapy programme (smartphone, tablet), which includes pelvic floor muscle training, bladder training, micturition diary and knowledge content for self-education. In the control arm, the previous treatments are continued. Patients in the control arm will then be given access to the digital therapy programme after the study is completed. The aim of the study is
Among other things, evidence of improvement in disease symptoms, quality of life and patient autonomy.

1.4 Study synopsis

Title of the study:	Prospective randomised study on multimodal self-treatment of women with incontinence complaints using a digital health application
Abbreviation of the study (acronym):	DINKS (Digital **Incontinence Study**)
Number of study centres:	1
EudraCT number:	2024-512094-27-00
EUDAMED-No. UDI-DI:	PP11745MICTERA96
Planned number of study participants:	198
Scientific management:	Professor Dr Axel Haferkamp<br>Clinic and Polyclinic for Urology and Paediatric Urology University<br>Medicine<br>Johannes Gutenberg University Mainz Langenbeckstraße<br>1<br>55131 Mainz
Diagnoses:	assigned to the female sex at birth ≥<br>18 years with incontinence of different (stress incontinence, urge incontinence, mixed incontinence)<br>ICD-10 codes:<br>- **N39.3 Stress incontinence [stress incontinence]**<br>- **N39.42 Urge incontinence**
Study design/methodology:	Randomised, controlled, 2-arm study, single-blind, monocentric.<br>Arm A: Intervention through digital therapy in addition to real-life care (Intervention group; multimodal self-treatment of incontinence)<br>Arm B: No access to digital therapy, access is granted after the end of the study, patient continues to have access to normal care reality (control group; waiting group design)<br>Duration of the intervention: 12 weeks

Goals of the clinical trial/objectives:	This superiority study is intended to demonstrate the following objectives:<br><br>Primary objective:<br><br>● Reduction in the frequency of incontinence episodes<br><br>(IEF) Secondary objectives:<br><br>● Improvement of disease symptoms in the ICIQ-SF<br>● Improving the quality of life in the I-QOL<br>● Improvement in the PAM-13 questionnaire (patient activation)<br>● Superiority in the PGI-I questionnaire (overall improvement after treatment)<br>● Increase in the proportion of patients without incontinence (cured patients)<br>● Reduction in micturition frequency during the day and at night<br>● Reduction of template consumption (pad use)<br>● Reduction of the imperative urge to urinate<br>● Improvement of functional bladder capacity<br>● Reduction in body weight<br>● Reduction of the share of patients with treatment failure<br>● Increase in the proportion of patients with a reduction in IEF from baseline to week 12 by at least 50%<br>● Reduction of the frequency of stress incontinence episodes in the S/U-IQ<br>● Reduction in the frequency of urge incontinence episodes in the S/U-IQ

Target values/criteria/endpoints:	Primary endpoint:<br><br>(1) Relative change (rel-CfB-IEF) in IEF from baseline to week 12 relative to baseline; desired reduction in mean relative IEF by 50% [6] [7]; IEF per 24 hours - averaged over 72 hours<br><br>Secondary targets / secondary target criteria:<br><br>(2) Change (CfB) in the ICIQ-SF from baseline to week 12; expected mean difference between the groups of≥ 2.9 points [8, 9]<br>(3) Change in I-QOL from baseline to week 12; expected mean difference between the groups of ≥ 6.3 points [9]<br>(4) Change in the PAM-13 questionnaire from baseline to week 12<br>(5) PGI-I questionnaire (overall improvement after treatment) at week 12<br>(6) Share of the patients without incontinence (cured patients) at the end of the study<br>(7) Change in micturition frequency per day from baseline to week 12<br>(8) Change in micturition frequency at night from baseline to week 12<br>(9) Change in pad consumption from baseline to week 12<br>(10) Change in imperative urge to urinate from baseline to week 12<br>(11) Change in functional bladder capacity from baseline to week 12<br>(12) Change in body weight from baseline to week 12<br>(13) Proportion of patients with treatment failure within the study period<br>(14) Proportion of patients with at least 50% reduction in IEF from baseline to week 12<br>(15) Change in stress incontinence episodes of the S/U-IQ from baseline to week 12<br>(16) Change in urge incontinence episodes of the S/U-IQ from Baseline at week 12

number of patients:	Randomised: Arm A to Arm B in a 1:1 ratio<br><br>Planning of study size (case number planning)<br><br>1. Relative reduction in incontinence episodes (rel-CfB-IEF), desired improvement of 50%, Minimum Important Difference (MID) approx. 45% from Yalcin 2010; significance level α= 5% 2-sided and Power 1 - β= 90%: N= 2 x 44= 88;+ 30% due to drop outs and their conservative imputation as well as subgroup analyses:<br>N= 120<br><br>2. **ICIQ-SF**: d= 2.9 MID from Nyström et al., 2015 and Asklund et al., 2017<br><br>Significance level α = 5% 2-sided and power 1 - β = 90%: N = 2 x 44= 88;+ 20% due to drop outs and their conservative imputation: N = 110<br><br>3. **I-QOL**: d= 6.3 (MID) from Yalcin et al., 2006<br><br>Significance level α = 5% 2-sided and power 80%: due to drop outs and their conservative imputation:<br>N = 158 + 40= 198<br><br>4. **PAM-13**: CfB with desired improvement by 6 points vs. control (MCID = 6, i.e. approx. 15% of the PAM rank from 13 - 52 and from John, 2020) by d/s = 6/12 = 0.5 (with α = 5% 2-sided and power = 80%: N = 2 x 64.<br><br>Based on these calculations, a case number of 198 is planned.
Inclusion criteria:	● ICD-10 codes:<br><br>**N39.3 Stress incontinence N39.42 Urge**<br><br>**incontinence**<br><br>**Mixed incontinence**<br><br>● Persons assigned to the female sex at birth with urge incontinence, stress incontinence, mixed incontinence (IE ≥ 1 per day)<br>● Age≥ 18 years<br>● Mastery of the German language<br>● Internet access<br>● Tablet or smartphone access (Android not older than version 7 and iPhone not older than iPhone 9)<br>● Informed Consent: Written consent of the participating patients

Exclusion criteria:	1. Absolute exclusion criteria that can be coded according to ICD-10 (incl. ICD-10 codes):<br>• None<br><br>2. Absolute exclusion criteria that cannot be coded according to ICD-10:<br>• None<br><br>3. Relative exclusion criteria that can be coded according to ICD-10 (incl. ICD-10 codes):<br>• Acute cystitis N30.0<br>• Urinary bladder calculi N21.0<br>• Conservative not controllable, recurrent macrohaematuria R31<br>• Newly diagnosed bladder carcinoma< 3 months C67<br><br>4. Relative exclusion criteria that cannot be coded according to ICD-10:<br>• Inability physically participate in the to participate in the therapy programme<br>• Active pregnancy<br>• Botulinum toxin treatment of the urinary bladder in the last 6 months<br><br>5. Study-specific methodological exclusion criteria:<br>• Generally patients who are unable to understand and sign the consent form independently<br>• Newly initiated drug or physical therapy for micturition symptoms (anticholinergics, beta 3 receptor agonists, phytopharmaceuticals, biofeedback, EMS) in the last 4 weeks<br>• Severe psychiatric disorders that make it impossible to use the app<br>• Neurological diseases that affect the urinary tract<br>• Tumour and incontinence operations in the small pelvis<br>• Obesity per magna (BMI >35)<br>• Recurrent urinary tract infections N39.0 (at least 2 within 6 months or 3 infections within one year)<br>• Participation of the patient in another clinical trial within the last 4 weeks prior to the Inclusion

	• addiction or other illnesses that do not allow the person concerned to assess the nature, scope and possible consequences of the clinical trial
Treatments/procedures Treatment plan (incl. aftercare):	The digital therapy to be tested via app contains the following components:<br>• Micturition diary<br>• Pelvic floor training and physiotherapy exercises<br>• Bladder training / cognitive behavioural therapy<br>• Mental exercises<br>○ Mindfulness<br>○ Progressive muscle relaxation (PMR)<br>○ Stimulus control methods<br>• Acute urge control<br>• Knowledge section / Nutrition section<br>• Progress tracking and motivation<br>The therapy instructions are available at via a smartphone.<br>/tablet-based app in the form of audio, video and text. The medical benefit is assessed via self-reported data points and validated questionnaires from the baseline survey compared to the end of the survey after 12 weeks.
Timetable (duration of study):	<u>Patient-centred:</u><br>Duration of study-related measures: 12 weeks<br>follow-up period: 12 months<br><u>Study-related:</u><br>Recruitment phase: 01.04.2024 to 31.10.2024<br>Start date: 01.04.2024<br>Treatment duration: 12 weeks<br>End of study: 15.02.2025<br>Final report: 15.04.2025
Test centres:	Clinic and Polyclinic for Urology and Paediatric Urology University<br>Medicine<br>Johannes Gutenberg University Mainz Langenbeckstraße<br>1<br>55131 Mainz

Docusign Envelope ID: 05A6509E-2636-42E1-8D3B-8B2D12CE3674

Statistical methods:	The primary endpoint (1) relative change rel-CfB-IEF week 12 regarding baseline of incontinence episodes is analysed using an analysis of covariance with the baseline value of the IEF as covariate and the factor treatment; the significance level is 5% 2-sided. Endpoint (2) change in the ICIQ-SF questionnaire, endpoint (3) change in quality of life in the I-QOL questionnaire and endpoint (4), change in PAM-13, are tested analogously with an ANCOVA at the 5% 2-sided level; to control for multiplicity, the analyses of endpoints (1) - (4) are tested sequentially using the method of a priori ordered hypotheses.<br><br>The other endpoints are analysed descriptively with p-values and 95% CIs.
Financing/Sponsor:	Kranus Health GmbH

1.5 Business round scheme

Table 1: *Visit scheme for the DINKS study*

				Study implementation / treatment phase					Follow Up		
	Before enrolment/ pre-screening	Inclusion	Baseline/telephone V1 week 1*	Onboarding IG/ after study inclusion	Technical support call week 2	V2 Week 6*	End of study Telephone V3 week 12*	Onboarding KG/IG	Follow-up questionnaire week 24* (IG, KG)	Follow-up questionnaire week 36* (IG, KG)	Follow-up questionnaire week 48* (IG, KG)
Medical history/basic data collection	X	X									
Stress-Urge-Incontinence-questionnaire (S/U-IQ)			X				X		X	X	X
Verification of input and output Exclusion criteria	X	X									
Clarification, Declaration of consent		X									
Randomisation			X								
Questionnaires (I-QOL and ICIQ-UI-SF)			X				X		X	X	X
PGI-I							X		X	X	X
Technical support				X (IG)	X (IG)			X			
Micturition diary				X (ongoing documentation possible)		X	X	X	X	X	X
Incontinence documentation over 72h		X					X		X	X	X
Acquisition (new) Concomitant medication/therapies	X	X	X			X	X		X	X	X
Recording of adverse events**		X	X	X	X	Additional telephone enquiry	X	X	X	X	X

*Week +/-7 d

**IA/SAE/Incidentes via SZ, by telephone (Tel: +49 89 380 38658) or by e-mail study-safety@kranus.de IG: Intervention group

KG: Control group

Docusign Envelope ID: 05A6509E-2636-42E1-8D3B-8B2D12CE3674

2 List of abbreviations

AE	Adverse event
Fig.	Illustration
AK	Exclusion criteria
AWMF	Working Group of the Scientific Medical Societies
BfArM	Federal Institute for Drugs and Medical Devices
BMI	Body mass index
CCS	Complete cases
CfB	Change from baseline
d	Day - Day(s)
DiGA	Digital health application
DINKS	Digital incontinence study (study acronym)
eCRF	electronic Case Report Form
ePRO	electronic Patient-Reported-Outcomes
EK	Inclusion criteria
EMS	Electrical muscle stimulation
FAS	Full Analysis Set
FPI	First Patient In
FPO	First Patient Out
GCP	Good Clinical Practice
ICD	International Classification of Diseases
ICH-E3	Structure and Content of Clinical Study Reports E3
ICH-GCP	International Conference on Harmonisation - Good Clinical Practice Guidelines
ICIQ-SF	Incontinence Questionnaire-Urinary Incontinence Short Form
I-QOL	Incontinence quality of life questionnaire
IEF	Incontinence episode frequency

Docusign Envelope ID: 05A6509E-2636-42E1-8D3B-8B2D12CE3674

ISO	International Organization for Standardisation
JtR	Jump-To-Reference
AI	Confidence interval
LOCF	Last Observation Carried Forward
LPI	Last Patient In
LPO	Last Patient Out
n. a.	Not applicable
NN	Nomen nominandum
NtF	Note to File
MDR	Medical Device Regulation
MID	Minimum Important Difference
OAB	Overactive Bladder
PAM-13	Patient Activation Measure 13
PGI-I	Patient Global Impression of Improvement -Incontinence
PMR	Progressive muscle relaxation
PPS	Per protocol set
Rel-CfB	Relative Change from Baseline (CfB/Baseline)
PROM	Patient reported outcome measures
QOL	Quality of Life
SAE	Serious Adverse Event
SAP	Statistical analysis plan
SAF	Safety Set
SF	Short Form
S/U-IQ	Stress/Urge Incontinence Questionnaire
V	Visit
vs.	Versus
WoC	Withdrawal of Consent

3 Test population

3.1 Inclusion criteria

- ICD-10 codes:

  N39.3 Stress incontinence N39.42 Urge incontinence

  Mixed incontinence

- Persons with urge incontinence who were assigned to the female sex at birth, Stress incontinence, mixed incontinence (IE≥ 1 per day)
- Age≥ 18 years
- Mastery of the German language
- Internet access
- Tablet or smartphone access (Android not older than version 7 and iPhone not older than iPhone 9)
- Informed consent: written consent of the participating patients

3.2 Exclusion criteria

Absolute exclusion criteria that can be coded according to ICD-10 (incl. ICD-10 codes):

- None

Absolute exclusion criteria that cannot be coded according to ICD-10:

- None

Relative exclusion criteria that can be coded according to ICD-10 (incl. ICD-10 codes):

- Acute cystitis N30.0
- Urinary bladder calculi N21.0
- Recurrent macrohaematuria that cannot be controlled conservatively R31
- Newly diagnosed bladder carcinoma< 3 months C67

Relative exclusion criteria that cannot be coded according to ICD-10:

- Inability to physically participate in the therapy programme
- Active pregnancy
- Botulinum toxin treatment of the urinary bladder in the last 6 months

Study-specific methodological exclusion criteria:

- Generally patients who are unable to understand and sign the consent form independently

Docusign Envelope ID: 05A6509E-2636-42E1-8D3B-8B2D12CE3674

- Newly started drug or physical therapy for micturition symptoms (anticholinergics, beta-3 receptor agonists, phytopharmaceuticals, biofeedback, EMS) in the last 4 weeks
- Severe psychiatric disorders that make it impossible to use the app
- Neurological diseases that affect the urinary tract
- Tumour and incontinence operations in the small pelvis
- Obesity per magna (BMI >35)
- Recurrent urinary tract infections N39.0 (at least 2 within 6 months or 3 infections within one year)
- Participation of the patient in another clinical trial within the last 4 weeks prior to inclusion
- addiction or other illnesses that do not allow the person concerned to assess the nature, scope and possible consequences of the clinical trial

Both groups, intervention and control group, have access to the treatment options of the regular care reality during the study period. In order to be able to evaluate the effect of the intervention in a differentiated manner, ongoing incontinence treatment (e.g. medication, interventional or surgical measures) should have been started at least four weeks before the start of the study. New therapies before the start of the study are considered an exclusion criterion.

During the study period, all newly initiated therapies for incontinence treatment (e.g. medication, interventional or surgical measures) are recorded and categorised as treatment failure. Treatment failure is also deemed to have occurred if the patient reports a deterioration in the PGI-I questionnaire at the end of the study.

The number of treatment failures is calculated and documented separately for the intervention and control groups.

Patients in the control group are not given access to the app-based intervention during the twelve-week study period. After completion of the survey at the end of the study (week 12, see 1.5. Visit schedule), they are free to use the app-based therapy.

4 Question and background

Urinary incontinence in women is a very common, often under-diagnosed and taboo condition, the main symptom of which is uncontrolled urine loss. This is accompanied by a significant reduction in quality of life and considerable suffering. Incontinence complaints are divided into stress incontinence, urge incontinence and mixed urinary incontinence.

4.1 Forms of urinary incontinence

4.1.1 Stress incontinence

Stress with an increase in intra-abdominal pressure (lifting, coughing, sneezing, laughing, jumping, etc.) leads to involuntary loss of urine, which is known as stress incontinence. One of the causes is a decrease in tone or pre-damaged pelvic floor muscles, for example after pregnancy and childbirth or in old age as a result of sarcopenia and decreasing muscle strength.

The severity of stress incontinence is determined by the intensity of the stress:

Grade 1: Leakage of urine when coughing, sneezing, pressing, lifting, carrying heavy objects.

Grade 2: Urine leakage when walking or standing up.

Grade 3: Urine leakage even when lying down.

4.1.2 Urge incontinence

Urge incontinence is when there is an involuntary loss of urine combined with a sudden urge to urinate (imperative urge to urinate). This is caused by involuntary spontaneous contractions of the detrusor of the bladder (detrusor hyperactivity, OAB overactive bladder).

4.1.3 Mixed incontinence

Mixed incontinence is when there involuntary loss of urine during physical exertion on the one hand and an imperative urge to urinate in the sense of urge incontinence on the other. In most cases, one of the components predominates.

4.2 Distribution and frequency

According to clinical studies, the distribution is as follows: Stress incontinence (50%), urge incontinence (14%), mixed form (32%) [10].

In Germany, around 15-25% of women are affected by various forms of urinary incontinence [1]. The frequency increases age. A high number of unreported cases can be assumed [2].

Docusign Envelope ID: 05A6509E-2636-42E1-8D3B-8B2D12CE3674

4.3 Diagnosis of urinary incontinence

The diagnostic recommendations for urinary incontinence in the German S2 guidelines are based on expert opinion and clinical studies [2]. The diagnostic steps are outlined below:

A detailed **medical history** enables incontinence to be categorised in the majority of cases [11]. In addition to the obstetric and gynaecological history, concomitant illnesses and medication are also relevant. The patient's individual impairment and level of suffering should also be enquired about.

The general **clinical examination** serves to rule out high residual urine volumes, resistance or masses in the pelvis. The gynaecological examination can be used to assess possible diseases of the external genitalia and a prolapse of the internal genitalia (descensus, prolapse). Palpation of the pelvic floor muscles enables an assessment of their contractility.

The use of validated **questionnaires** is recommended in guidelines. The severity of the disease, type of incontinence and disease-related quality of life can be recorded.

The **micturition diary** is used to quantify symptoms and record the frequency of incontinence episodes. It also enables the measurement of micturition volumes and the determination of the 24-hour total urine volume or the nocturnal urine volume. The use of the micturition diary is strongly recommended in the guidelines for all forms of incontinence [2]. To increase the accuracy of the results, the use of a three-day micturition diary is used to determine a 24-hour average [12].

The **pad test** quantifies urine loss by wearing pads during documented physical activity and weighing them after a set period of time. Both the 1-hour pad test and the 24-hour pad test exist. Although the 24-hour test is more precisely reproducible, the standardisation of physical activities is more complex [13].

It is important to know if there is **residual urine** before initiating treatment for incontinence, as drug therapy options such as anticholinergics can impair bladder emptying. The amount of residual urine is determined by ultrasound examination. However, studies on the determination of residual urine in women are rare and there is no standard definition for a pathological amount of residual urine. In women with stress incontinence, only 16% had residual urine > 100 ml [14].

Urodynamics with bladder manometry, uroflowmetry and urethral pressure profile are used to assess the bladder during the filling and emptying phase. Routine use of this examination prior to conservative treatment of uncomplicated stress incontinence and untreated overactive bladder is not recommended in the guidelines [2].

Of the **imaging procedures**, sonography plays an important role in determining residual urine (see above). Transcavitary functional sonographic procedures are only relevant in the context of surgical treatment of incontinence.

To summarise, it can be stated that, of the diagnostic procedures described, only questionnaires and pad tests for the quantitative assessment of the

Docusign Envelope ID: 05A6509E-2636-42E1-8D3B-8B2D12CE3674

incontinence and to test the effectiveness of therapy. However, the use of the various validated questionnaires is heterogeneous [4].

4.4 Treatment of urinary incontinence

In the clinical practice of incontinence treatment, **conservative therapies** (less invasive, reversibility) are generally used before surgical therapies. For all types of incontinence, pelvic floor muscle training, cognitive behavioural therapy, lifestyle changes and self-management therapies are successful and represent an alternative to drug therapies [15]. For this reason, both the German AWMF guidelines [2] and the European guidelines for urology (EAU guidelines) recommend these measures with strong scientific evidence [3].

4.4.1 Underlying diseases and medication

Existing underlying diseases (e.g. diabetes, heart failure, obesity, neurological disorders, etc.) can have an impact on incontinence and should be treated appropriately. The influence of obesity as a risk factor for urinary incontinence has been demonstrated [16]. Studies on the therapeutic effect of weight reduction show contradictory results [2].

The influence of medication for comorbidities on urinary incontinence is difficult to assess and the benefit of adapting drug therapies to improve incontinence has not been proven [2].

4.4.2 Lifestyle changes

Lifestyle factors that can be associated with incontinence include obesity, smoking, lack of physical activity, drinking habits and diet. Adjusting these factors can improve urinary incontinence [2].

4.4.3 Physiotherapy/ pelvic floor training

Pelvic floor exercises are used to improve the function of the pelvic floor and urethral stability.

Meta-analyses have shown that pelvic floor muscle training is effective in terms of curing and relieving urinary incontinence and improving the quality of life of those affected [4] [15] [17] [18]. These effects apply to women with stress incontinence, urge incontinence and mixed urinary incontinence [19].

Improved pelvic floor function can inhibit non-physiological bladder contractions in patients with an overactive bladder, leading to an improvement in symptoms [20]. Pelvic floor training also increases the likelihood of continence 12 months after birth in cases of urinary incontinence caused by pregnancy and childbirth [21]. In addition, older women with stress incontinence benefit from pelvic floor muscle training just as much as younger women [22] [23].

Accordingly, the guidelines recommend carrying out pelvic floor muscle training for both urge and stress incontinence for a period of at least three months [2].

Docusign Envelope ID: 05A6509E-2636-42E1-8D3B-8B2D12CE3674

4.4.4 Bladder training

Through bladder training, the patient learns to better assess the filling status of the bladder and not to "preventive" emptying of the bladder. Gradually adapted voiding intervals are built up. The specific aims are to correct incorrect habitual patterns, gain control over urge incontinence and ultimately extend the voiding intervals by increasing functional bladder capacity. Incontinence episodes can thus be reduced and patients' self-confidence in their ability to control their bladder function independently is strengthened [2].

4.4.5 Drug therapy

Antimuscarinic drugs are a relevant option in the secondary (after failed non-medication therapy) treatment of overactive bladder and urge incontinence. Although the available drugs differ in their pharmacological profiles, what they all have in common is their inhibitory effect on the muscarinic receptors of the detrusor cells. This leads to a reduction in spontaneous, uncoordinated detrusor contractions [2]. The efficacy of antimuscarinic drugs has been proven in numerous studies (overview e.g. in [24]).

Side effects include dry mouth, constipation, accommodation disorders of the eyes, cognitive impairment, tachycardia and fatigue. There is a relatively high discontinuation rate with the main causes being ineffectiveness, side effects and the ability to manage symptoms without medication [2].

The ß3-adrenoreceptor agonist **mirabegron** has also shown efficacy in clinical trials [25]. The side effect profile differs from that of antimuscarinics, whereby the risk of arterial hypertension in particular should be taken into account. However, mirabegron does not lead to any impairment of bladder contractility [2].

Duloxetine prevents the presynaptic reuptake of the neurotransmitters serotonin and noradrenaline. This results in increased stimulation of the 5-hydroxytryptamine and noradrenaline receptors on motor pudendal nerves. This leads to increased resting tone and increased contraction force at the striated urethral sphincter [2]. The results from clinical studies are heterogeneous. The German S2 guidelines recommend that women with moderate urinary incontinence and above should be informed about duloxetine as a treatment option [2].

4.4.6 Digital therapy programmes

There are currently no digital health applications for the treatment of female incontinence listed on the BfArM website in Germany (https://diga.bfarm.de/de/verzeichnis). However, self-management programmes using smartphones or tablets are listed in the literature. For example, a randomised controlled trial on the effectiveness of an app-based intervention for female urinary incontinence through pelvic floor muscle training, bladder training and knowledge transfer showed after four months that the app therapy was as effective as guideline-based care by general practitioners in the Netherlands [26]. Both treatments led to a relevant reduction in urinary incontinence symptoms, improvement in quality of life and fewer daily incontinence episodes. The authors conclude that app-based therapy is a comparably effective but more accessible alternative to conventional care [26].

Docusign Envelope ID: 05A6509E-2636-42E1-8D3B-8B2D12CE3674

A systematic review that investigated the intervention of pelvic floor training using an app in women with stress incontinence or stress-related mixed incontinence using six randomised intervention studies found that app-based pelvic floor training significantly reduced the severity of symptoms of stress incontinence in the ICIQ-SF questionnaire and improved the quality of life of the women affected [27]. This demonstrates the effectiveness of app-based pelvic floor exercises for the treatment of stress urinary incontinence and for improving the quality of life of affected women. The results of the Patient Global Impression of Improvement (PGI-I) at follow-up of the studies analysed indicate that participants in the app group experienced a significant improvement [27]. This systematic review focuses on apps that only offer pelvic floor muscle training as an intervention for stress urinary incontinence. A digital health application (DiGA) that integrates other elements of urinary incontinence therapy in addition to pelvic floor muscle training, such as lifestyle changes, fluid management and bladder training - in line with German and European guidelines - could potentially increase effectiveness. A multimodal therapy approach could therefore increase the likelihood of sustainable therapeutic success.

4.5 Conclusions and rationale for the study

Urinary incontinence in women is a very common condition that can be categorised as stress, urge or mixed urinary incontinence. There are various treatment options for different forms of incontinence, including lifestyle changes, physiotherapy/pelvic floor exercises, micturition monitoring and also medication and surgical procedures.

Despite the recommendation in the guidelines [2, 3] and proven effectiveness [4] as a primary therapy, comprehensive, multimodal therapy and monitoring programmes (bladder diary, pelvic floor training, lifestyle changes, fluid management, bladder training, etc.) for female incontinence are rarely offered in clinical practice. The reason for this is the complexity of organisation and implementation in the "analogue" world. Although the treating physician has contact persons (e.g. physiotherapists) available for the individual modules, close and cross-module monitoring of patients, which is essential for the success of treatment [2, 19], cannot be realised in clinical practice. A DiGA can help to close a gap in care by monitoring, treating and alleviating urinary incontinence in line with the areas of application defined in the Digital Healthcare Act.

The positive care effect of such a DiGA is to be demonstrated in a randomised study for patients with stress incontinence (N39.3) and urge incontinence (N39.42) as well as mixed forms. In the intervention arm, patients will have access to a 12-week digital therapy programme based on evidence-based approaches and including measures recommended by German and European guidelines for the treatment of incontinence (see 4.4 Treatment of urinary incontinence). This therapy programme specifically includes pelvic floor training, bladder training, keeping a micturition diary and informative sections on knowledge transfer and education. Previous treatments can be continued in the control arm, i.e. control group patients continue to have access to the reality of care. During the study period, all newly initiated incontinence treatment therapies (e.g. medication, interventional or

Docusign Envelope ID: 05A6509E-2636-42E1-8D3B-8B2D12CE3674

surgical measures) and categorised as treatment failure. The aim of the study is, among other things, to prove the improvement of disease symptoms, quality of life and patient autonomy.

Docusign Envelope ID: 05A6509E-2636-42E1-8D3B-8B2D12CE3674

5 Study endpoints and supply effects

The aim of this study is to test a multimodal digital therapy via app for women with stress incontinence, urge incontinence and mixed urinary incontinence. The assessment of the positive effects of the treatment is based self-reported documentation by the study patients and validated questionnaires (see appendix).

5.1 Incontinence episodes frequency (IEF)

To increase the accuracy of the results, the incontinence episodes are recorded over a period of 72 hours and an average is calculated over 24 hours [12]. This average value per 24 hours is used as the IEF for the assessment of the primary endpoint. The aim is a relative reduction in incontinence episodes (rel-CfB-IEF) of 50% in the app group compared to the control group.

5.2 Questionnaires

The standardised assessment of urinary incontinence is carried out using questionnaires validated German (see

Table 2). All of the incontinence-specific questionnaires listed below that were selected to record the secondary endpoints of this study are considered Category A questionnaires according to the guidelines and therefore fulfil the criteria for validity, reliability and the recording of changes [2] .

***Table 2**: Questionnaires*

Questionnaire	Purpose
ICIQ-SF	Incontinence symptoms
I-QOL	Quality of life
PAM-13	Self-management skills
PGI-I	Overall improvement
S/U-IQ	Distinction between stress and Urge incontinence

5.2.1 ICIQ-SF questionnaire

The International Consultation on Incontinence Questionnaire - Short Form (ICIQ-SF) is a standardised questionnaire to assess urinary incontinence in urology. It was developed to assess the frequency and severity of urinary incontinence and its impact on the quality of life of those affected. The ICIQ-SF comprises four questions, three of which are used to calculate a score with a maximum score of 21 points. The fourth question is asked to determine the urinary leakage situation. Clinically relevant urinary incontinence is defined as four points or more. The German S2 guidelines for urinary incontinence recommend the use of the ICIQ-SF questionnaire to assess the severity of urinary incontinence and to measure changes during the course of interventions. In the study described here, this questionnaire is at the beginning (baseline) and after 12 weeks. This endpoint is

Docusign Envelope ID: 05A6509E-2636-42E1-8D3B-8B2D12CE3674

is achieved if the change in the ICIQ-SF from baseline to week 12 is > 2.9 points [8, 9].

5.2.2 I-QOL questionnaire

The Incontinence Quality-of-Life (I-QOL) is a validated questionnaire recommended by the German guidelines for urinary incontinence, which has been validated for both stress and urge incontinence and assesses the impact of urinary incontinence on quality of life. This questionnaire specialises in assessing the quality of life associated with incontinence problems. The I-QOL questionnaire takes into account emotional, social and physical aspects. Higher scores on the I-QOL indicate a better quality of life, while lower scores indicate greater impairment due to urinary incontinence. There are often specific sub-areas that represent individual aspects of quality of life and scoring can also be done separately for these areas. To assess the change in quality of life, this questionnaire is completed at baseline and after 12 weeks. The endpoint is reached when the change in I-QOL from baseline to week 12 is > 6.3 points [9].

5.2.3 PAM-13 Questionnaire

The Patient Activation Measure-13 (PAM-13) is a validated questionnaire designed to measure patients' level of activation in relation to their healthcare. The PAM-13 was developed to assess patients' self-competence and knowledge about their own healthcare. The questionnaire consists of 13 questions that relate to different aspects of patient activation. The questions can cover topics such as understanding of health information, ability to self-organise and willingness to actively participate in their own healthcare. The questionnaire uses a Likert scale anchored between 1 and 4 (1=disagree, 2=somewhat disagree, 3=somewhat agree, 4=strongly agree); the range is from 13 - 52 score points; it is transformed to a scale of 0-100, with higher scores a higher level of activation. Four activation levels are distinguished, reflecting low, basic, moderate and high activation. The PAM-13 enables the assessment of empowerment and the self-management skills of patients. This is completed at the beginning and after 12 weeks.

5.2.4 PGI-I questionnaire

The PGI-I questionnaire stands for "Patient Global Impression of Improvement". It is used to record patients' subjective assessment of the improvement in their state of health or symptoms. The questionnaire consists of a single question that asks patients to give their own assessment of the improvement in their state of health. Using the PGI-I questionnaire, patients can rate their health status from "much better" to "much worse". In research and clinical practice, the PGI-I questionnaire is used to take the patient perspective into account when evaluating the effectiveness of interventions. In the study described here, this questionnaire is completed at the end of the study [28].

5.2.5 S/U-IQ questionnaire

The stress/urge incontinence questionnaire is a validated questionnaire that consists of two questions and makes it possible to differentiate between stress and urge incontinence. In the case of mixed incontinence, this questionnaire helps to determine the dominant incontinence component [29]. It is completed at the beginning and after 12 weeks.

5.2.6 Further questions about app use

In week 12, the intervention group receives the following additional questions, which the patient can answer voluntarily:

- Please select the number that best describes the condition of your incontinence now compared to before the therapy: A scale of 1-7 (1 much better - 7 much worse) is provided here.
- How often have you accessed Mictera? (Several times a week, weekly, with longer breaks than 1 week, with longer breaks of between 2-6 weeks, with longer breaks of over 6 weeks, I have not used Mictera)
- If you took longer breaks or did not train with Mictera, what was the reason for this? (free text)
- How are you to recommend Kranus Mictera to others? Answer on a scale of 1-10 (1 Not at all - 10 Very much)
- How satisfied are you with the app? Answer on a scale of 1-10 (1 Not at all - 10 Very much)
- Do you have any comments or suggestions for us? The answer is given here in free text.

The control group is only asked one further question in week 12:

- Do you have any comments for us? This question can be answered in a free text field.

Both groups are asked questions in week 1:

- Please your year of birth.
- Please enter your current weight. (in kg)
- How tall are you? (in cm)

5.3 Therapy failure

Treatment failure is defined as any reported worsening in the PGI-I questionnaire at the end of the study or the start of any interventions against the incontinence symptoms (conservative and invasive methods).

Docusign Envelope ID: 05A6509E-2636-42E1-8D3B-8B2D12CE3674

6 Study design and description

6.1 Type of study, therapy allocation, scope of the study

This is a single-blind, two-arm, randomised study with an intervention and a control group. The study is being conducted at Mainz University Hospital and recruitment is taking place throughout Germany. A total of 198 patients will be included, 99 in the intervention group and 99 in the control group.

6.2 Patient recruitment

The study centre is a urological university clinic. Patients recruited via the study centre as well as via urological, general practitioner or gynaecological outpatient clinics and practices throughout Germany and via direct contact with affected patients.

6.3 Timetable

Recruitment phase: 01.04.2024 to 31.10.2024 Study start:

with positive ethics vote (FPI) Treatment duration: 12 weeks.

End of studies: 15 February 2025 (LPO)

Final report: by 15 April 2025

6.4 Study centre

The responsible study centre is the Urological University Clinic Mainz. The centre receives training on the study and the corresponding information and training documents. The training is documented in writing. The centre receives the complete study documents.

6.5 Study programme

The study will be recruited via the Kranus Health GmbH network of gynaecologists, general practitioners and urologists, by directly approaching patients, a study landing page and, if necessary, online advertising. **Figure 1** outlines the study process.

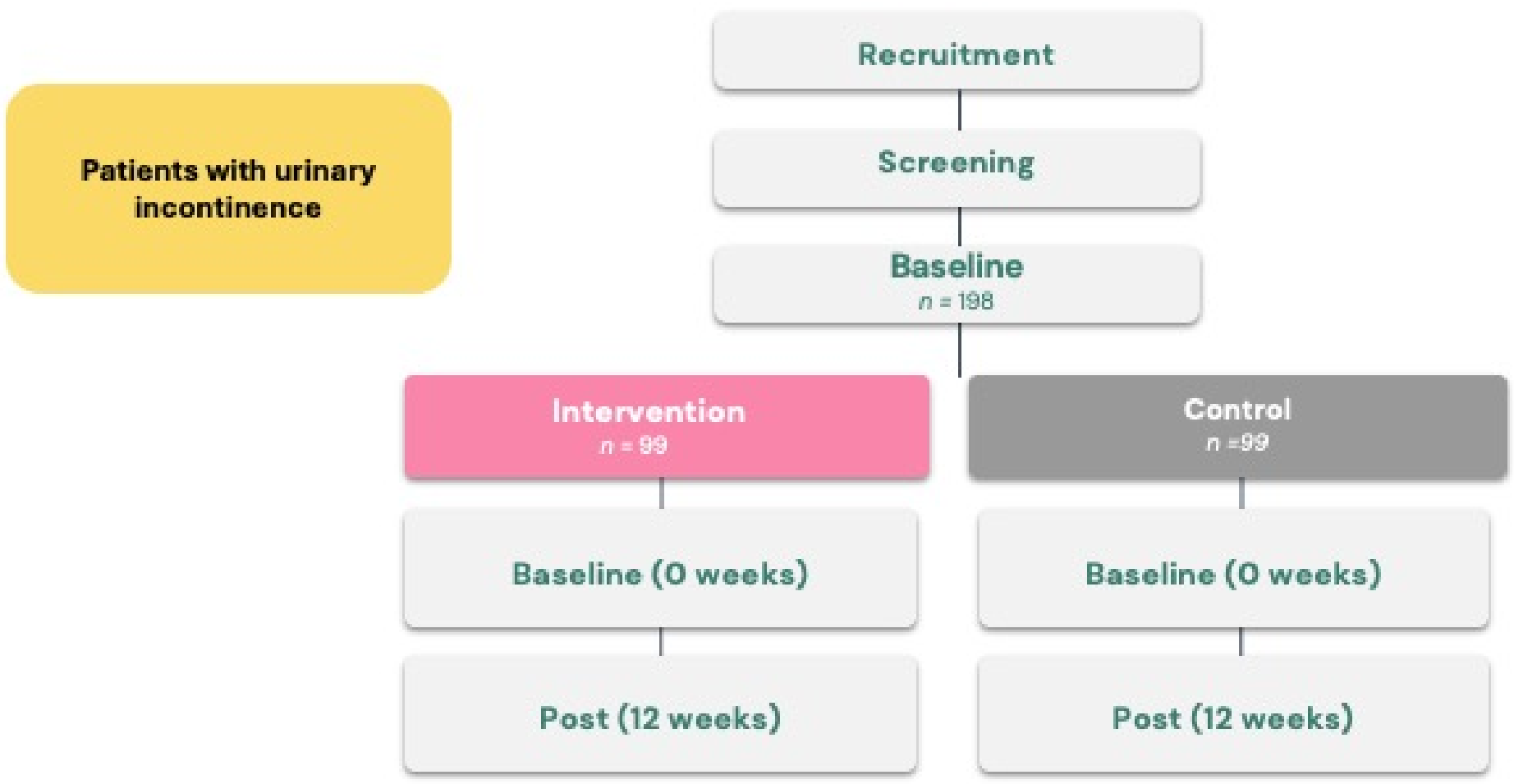

Figure 1: *Study flow chart*

The end of the programme is planned for 12 weeks after LPI.

6.6 Patient insurance

During the study, all study participants are insured in accordance with the applicable law. According to the General Terms and Conditions of Insurance, the insurance cover extends to all damage to health that occurs as a result of the study during and after participation. Only financial disadvantage is covered; no compensation for pain and suffering is paid.

6.7 Screening, information and consent

The initial screening of patients takes place via the study portal and urological, gynaecological and general medical practices. If patients are interested in participating in the study, they register in the study tool (Dacima from Evident IQ, information on data security and compliance of the server structure is provided in accordance with ISO27001 and specified in the other documents attached to the submission) and are assigned a patient ID. The study centre in Mainz will check the inclusion and exclusion criteria and, if necessary, subsequently inform the patients. The study participant will be informed about the nature, significance, objectives, possible risks, expected benefits, scope and other aspects of the study.
After the information session, each patient is given sufficient time and opportunity to clarify any unanswered questions and decide whether to participate.

Each eligible patient signs and dates her consent to participate in the study, voluntarily, in person on the consent form online or by hand. The patient's consent also expressly refers to the collection and processing of personal data.

Docusign Envelope ID: 05A6509E-2636-42E1-8D3B-8B2D12CE3674

data. Patients are therefore explicitly informed about the purpose and scope of the collection and use of this data, in particular health data.

One copy of the signed consent form (copy or 2nd original) remains with the patient or is sent by email, the other in electronic form on the study server, to which only the study centre has access.

The patient can withdraw consent at any time and without giving reasons and discontinue treatment or end the study. In such cases, the patient is asked to state the reason for withdrawal (for treatment or participation), but is advised that she does not have to do so. The time of withdrawal of the declaration of consent for the study must be documented.

The patient information and consent form is a separate document to this study protocol.

Patients for whom it becomes apparent during the informed consent discussion with the study centre that they do not meet an inclusion criterion, meet an exclusion criterion or for whom it becomes apparent after signing the informed consent form that they do not meet the eligibility criteria for participation in the study are assessed and documented as screening failures.

6.8 Pre-screening, medical history, inclusion procedure

In the first step, the prospective patient registers via the study portal and fills out a few questions to initially check potential eligibility based on the inclusion and exclusion criteria. If the result of the check is positive, the patient registers in Evident IQ's Dacima study tool, receives a patient ID, fills out a medical history form and provides proof of diagnosis. If the criteria are met, an appointment is made with the study centre, which checks the inclusion and exclusion criteria again. The patient is then informed about the study and can ask questions about the study and its procedure. Once the consent form has signed voluntarily and independently, the patient is included in the study.

Essential basic information (study centre, date of consent, year of birth, age, gender, inclusion/exclusion criteria, etc.) is stored in the eCRF. All patients record their incontinence episodes over a period of three days (3 x 24 h). The baseline data collection then takes place in the study tool, followed by randomisation to the respective study arm in the study tool. For documentation purposes, a form on the randomisation result (block randomisation 1:1) is generated via the study tool. Furthermore, the intervention patients fill out a micturition diary in the app to record micturition frequency, drinking volume and other symptoms.

6.9 Treatment phase

During the treatment phase, the patient is required to log into the Mictera app regularly and carry out the activities described for the day in question. Compliance with the set therapy goals is monitored via the Mictera app in pseudonymised form and reminders are communicated to the patient if necessary. The activity of app use is automatically documented.

The therapy is based on the principle of digital content, which includes personalised multimodal therapy with a focus on pelvic floor training, physiotherapy, mental training and

Docusign Envelope ID: 05A6509E-2636-42E1-8D3B-8B2D12CE3674

behavioural therapy with methods of urinary urgency control and relaxation techniques. Another key component is the micturition diary, which patients can use to document their micturition behaviour. The approach includes several therapy programme elements whose effectiveness in the treatment of incontinence has been proven in the literature.

6.9.1 Micturition diary

Patients can document the amount they drink, meals, amount and frequency of micturition and symptoms. An algorithm helps with patient-individualised information. The visually simplified presentation helps patients to recognise correlations and motivate them.

6.9.2 Pelvic floor and physiotherapy exercises

The daily training consists of exercises focussing on the ability to repeatedly contract the pelvic floor muscles relevant for the function of the sphincter and the bladder reflex. The exercises are taught with the help of text, video instructions and interactive software elements. Special physiotherapy exercises for training the pelvic floor are suggested once a week. They are aimed at improving and supporting the pelvic floor. Here too, the exercises are taught with the help of text and detailed video instructions.

6.9.3 Bladder training / cognitive behavioural therapy

Behavioural therapy exercises are used to increase functional bladder capacity, reduce micturition frequency and urge problems. The patient therefore receives new content and goals in the app every week.

6.9.4 Mental exercises

- Mindfulness: Every week, patients receive new content on mindfulness-based approaches based on cognitive behavioural therapy strategies.
- Progressive muscle relaxation (PMR): The deliberate and conscious tensing and relaxing of certain muscle groups leads patients into a state of deep relaxation.
- Stimulus control methods: Unwanted thoughts such as the acute urge to urinate are acutely suppressed.
- All methods help patients to manage their symptoms and break through urge or urge conditioning. The content is provided in the form of audio sequences.

6.9.5 Acute urge control

In the event of a strong urge to urinate, the patient is given access to short versions of the structured exercises for mindfulness, PMR and thought control. They can also access a short pelvic floor training exercise or select a game to provide a situational distraction from the issue and gain control over the micturition reflex.

Docusign Envelope ID: 05A6509E-2636-42E1-8D3B-8B2D12CE3674

6.9.6 Knowledge section /Nutrition section

Users receive daily educational content on the causes and underlying principles of incontinence. Another focus is on nutrition-specific correlations. General therapeutic measures are explained as well as psychological, habitual and organic correlations.

6.9.7 Further functions:

Participants have the opportunity to monitor the course of their symptoms and the progress of their therapy and are motivated by receiving progress awards.

6.10 Visits and analyses

During the 12-week treatment phase, no routine visits to the study centre are planned. However, if necessary, the patient can contact her gynaecologist, urologist, etc., the study centre or the study hotline at any time.

The study-specific surveys are listed in the ward round schedule (see **Table 1:** ward round schedule for the DINKS study).

6.11 End of study participation

The regular end of study participation for each study participant is after 12 weeks and completion of the questionnaire in week 12. Participation in the study is voluntary. Each participant has the right to withdraw from the study at any time at her own request, prematurely and without giving reasons (consent can also be withdrawn in this case) or to decide to discontinue treatment (no further treatment) without any disadvantages for her further (medical) treatment. In a follow-up period of 12 months after the primary survey period, the patient has the opportunity to take part in further surveys. Patients in both groups (arm A and B) are given the opportunity to continue using the app. During this time, the patient can contact her study centre, her treating gynaecologist, urologist or the study hotline at any time.

If a patient withdraws her consent and leaves the study, this constitutes a withdrawal from participation (WoC). If it is possible and the patient is willing to provide information, the study centre will attempt to out the reason for the premature termination of the study. The participant is asked to state the reason for discontinuation, but is advised that she does not have to do so. It will be documented when and, if applicable, why she withdrew her consent.

6.12 End of the study

The planned end of the study is immediately after LPO.

Docusign Envelope ID: 05A6509E-2636-42E1-8D3B-8B2D12CE3674

7 Adverse events

7.1 Complications and risks

Muscle and joint complaints due to the physical strain are possible side effects of the therapy programme. Serious injuries should not occur due to the exclusion criteria.

7.2 Recording and documentation of adverse events

7.3 Adverse event

An "adverse event", hereinafter referred to as "AE", is an adverse medical event, inappropriate patient management decision, illness or injury, or adverse clinical symptoms, including abnormal laboratory findings, in study participants in the clinical trial, even if not related to the product tested in the clinical trial.

7.4 Serious Adverse Event

The SAE according to ICH-GCP (serious adverse event) or "serious adverse event" refers to an adverse event that had one of the following consequences:

1. a patient management decision that has resulted in death or an immediate life-threatening situation for the subject or the death of the subject's offspring;
2. Death;
3. serious deterioration in the subject's state of health, which in turn had one of the following consequences:
    a. life-threatening illness or injury;
    b. Permanent physical injury or permanent impairment of a bodily function;
    c. inpatient treatment or extension of the 's inpatient treatment;
    d. Medical or surgical intervention to prevent a life-threatening illness or injury or permanent physical damage or permanent impairment of a bodily function;
    e. chronic disease;
    f. foetal endangerment, death of the foetus or congenital physical or mental impairment or birth defects.

7.5 Product defect or device-related adverse events

The "product defect" means an inadequacy in the identification, quality, durability, reliability, safety or performance of the product, including malfunctions, application errors or inadequacy of information provided by the manufacturer and will also be reported, documented and evaluated to the sponsor.

Adverse events are documented in the eCRF by the study centre after consultation with the patient. In addition, patients are contacted by telephone once after six weeks to ask about potential SAEs and AEs.

Docusign Envelope ID: 05A6509E-2636-42E1-8D3B-8B2D12CE3674

All AEs and SAEs are documented in the eCRF in accordance with ISO14155. SAEs must be documented and transmitted to the sponsor within 24 hours of becoming known via the eCRF system (via eCRF and automatic e-mail from the eCRF to study-safety@kranus.de). The sponsor is then obliged to assess and report the SAE in accordance with its reporting obligations.

When using the Mictera therapy programme, the risk of undesirable side effects is generally very low. A comprehensive literature search was carried out for the authorisation as a medical device and for the study protocol. No relevant risks were described when carrying out the corresponding exercises.

7.6 Definition of the risk threshold

7.6.1 Risk threshold for patients

Patients should not have SAEs due to the use of Kranus Mictera or need to see a doctor due to this digital therapy.

For the study, a contact is made after 6 weeks within the 12-week therapy with Kranus Mictera as part of the monitoring of SAEs of study participants. If the patient reports an SAE during the course of the study, or if the patient has to see a doctor as a consequence of using Kranus Mictera, this is documented in the eCRF (including an automatic e-mail to study-safety@kranus.de) and the sponsor assesses whether there is a connection with the use of Kranus Mictera. If there is a connection with the digital therapy, will be evaluated whether further participation in the study is justifiable for the patient.

7.6.2 Risk threshold for the study

The study should not cause more than five identical or similar SAEs due to the use of Kranus Mictera.

The sponsor is informed of (S)AEs via eCRF andstudy-safety@kranus.de and monitors and assesses the SAEs that occur. If more than five SAEs occur, which firstly are directly related to the product and secondly have the same or a similar cause after assessment, it is assumed that the risk threshold for the study exceeded and the study is discontinued.

If necessary, the product "Kranus Mictera" can be withdrawn from the market immediately. In the event of serious risks, the sponsor shall also immediately inform the competent authorities and, where appropriate, the notified body that issued the certificate of conformity, providing in particular details of the non-conformity and of any corrective action already taken.

7.6.3 Onset of pregnancy

The occurrence of pregnancy is considered an adverse event (AE) in this clinical trial for safety reasons. If a pregnancy is reported, the patient will be excluded from the study as soon as it becomes known and access to the Kranus Mictera app will be withdrawn.

8 Statistics

8.1 Endpoints

The time point of interest for the target evaluation is after 12 weeks, i.e. the end of study for intervention arm A and the delayed start of the intervention in control arm B.

8.2 Primary endpoint

1) Relative change (rel-CfB-IEF) of the IEF from baseline to week 12 in relation to baseline

8.3 Secondary endpoints

2) Change in ICIQ-SF from baseline to week 12
3) Change in I-QOL from baseline to week 12
4) Change in the PAM-13 questionnaire from baseline to week 12
5) PGI-I questionnaire (overall improvement after treatment) at the end of the study
6) Proportion of patients without incontinence (cured patients) at the end of the study
7) Reduction in micturition frequency during the day
8) Reduction in micturition frequency at night
9) Reduction in pad consumption
10) Reduction of the imperative urge to urinate
11) Change in functional bladder capacity
12) Reduction in body weight
13) Reduction in the proportion of patients with treatment failure within the study period
14) Proportion of patients with at least 50% reduction in IEF from baseline to week 12
15) Reduction in the frequency of stress incontinence episodes in the S/U-IQ
16) Reduction in the frequency of urge incontinence episodes in the S/U-IQ

8.4 Planning the scope of the study (case number planning)

Randomised: Arm A to Arm B in a 1:1 ratio

Planning the scope of the study for comparisons between the groups:

- IEF: Relative reduction in incontinence episodes (rel-CfB-IEF) with desired improvement of 50%, Minimum Important Difference (MID) approx. 50% from Yalcin 2010; at d/s = 0.7 with significance level α = 5% 2-sided and power 1 - β = 90%: N = 2 x 44 = 88, + 20% due to drop outs and their conservative imputation as well as subgroup analyses: N = 120.

- ICIQ-SF: CfB with desired improvement by d = 2.9 vs. control (MID = 2.5 from Nyström 2014 and Asklund 2017) by d/s = 2.9/3.2 = 0.9 with α = 5% 2-sided and power 1 - β= 90%: N= 2 x 28.

- I-QOL: CfB with desired improvement by 6.3 vs. control (MID= 6.3 from Yalcin 2006) by d/s = 6.3/ 14 = 0.45 (with α = 5% 2-sided and power = 80%: N = 2 x 90.

- PAM-13: CfB with desired improvement by 6 points vs. control (MCID= 6, i.e. approx. 15% of the PAM rank from 13 - 52 and from John, 2020) by d/s = 6/12 = 0.5 (with α = 5% 2-sided and power = 80%: N = 2 x 64.

Due to drop-outs and their conservative imputation, the total number of cases is set at 2 x 99 study participants for the evaluation of the primary endpoints.

8.5 Discussion of the end points

Primary and secondary endpoints for urinary incontinence are not uniformly defined in the literature and are used heterogeneously in the various studies [30, 4, 31].

In principle, a distinction can be made between so-called subjective (based on questionnaires) and objective (number of incontinence episodes, pad tests) [32].

Both types of endpoints are used in studies as the primary "outcome variable" and accepted.

8.6 Primary endpoint

There is no standardised method for measuring incontinence episodes. In a study very similar to ours (app-based pelvic floor muscle training for stress incontinence), the incontinence episodes were measured in the micturition diary on 2 days and then multiplied by 3.5 to obtain a weekly average [8]. In a similar, internet-based pelvic floor training study, Sjöström et al. used an analogue measurement system [33]. In a study with different conservative measures (including pelvic floor muscle training), Bo et al. the incontinence episodes over 3 days [34]. Castro et al [35] measured incontinence episodes over 7 days in a similar study.

In the present study, incontinence episodes were measured over 3 days (baseline and end-of-study), the mean value per day was calculated and this was used as the primary endpoint.

There is a lack of data in the literature on the minimum clinically relevant difference (MID) in connection with specific measures such as medication or pelvic floor muscle training. In general, a reduction of 50% is described as clinically relevant for conservative measures for incontinence [6, 31]. Based on the literature, we define a reduction in incontinence episodes as the quantitative primary endpoint.

Statistically analysed is primarily the relative change (rel-CfB-IEF= CfB/B) of the IEF from baseline to week 12 in relation to baseline; desired reduction of the mean relative IEF by 50% versus control; IEF frequency= mean value of IEs over 24 hours calculated from IEs over 72 hours.

8.7 Secondary endpoints

The ICIQ-SF is used as a validated questionnaire [36] to assess the severity and impact of incontinence by the patient in several studies [30] [37].

An improvement of 2.5 score points is specified as the MID in Nystrom et al [38]. In the study by Sjöström [33] an improvement of 20% is aimed for, in the study by Asklund [8] an improvement of 2.9 points (based on the results of the Sjöström study) is described as the target (3.2 points were achieved in the study). Based on the various data in the

literature, we are aiming for an improvement of 2.9 points in the ICIQ-SF in this study (see **Table 3:** ICIQ-SF).

Table 3: *ICIQ-SF*

Tool/ Parameter	Questionnaire	Possible answers	Result range	Targeted improvement
ICIQ-SF	3	1. Question: 0-5<br>2. Question: 0,2,4,6<br>3. Question: 0-10	0-21	$\geq$ 2.9 points

As further secondary endpoints, the disease-related quality of life is measured using the validated I-QOL and the patients' level of activation in relation to their healthcare using the PAM-13 questionnaire, which has also been validated. The change in specific incontinence episodes, measured using the S/U-IQ questionnaire, is also recorded. Other endpoints include changes in micturition frequency during the day and night, reduction in pad use, decrease in imperative urge to urinate and an increase in functional bladder capacity as well as surveys on body weight changes, treatment failure and overall improvement (PGI questionnaire) and app use in the intervention group.

8.8 Statistical analysis

In general, continuous data is summarised using descriptive statistics, i.e. with the number of observations, mean, standard deviation, minimum, median and maximum. For some variables such as the primary endpoints, the change from baseline to the end of the study (change from baseline, CfB) is also output and summarised as "post-baseline". Where appropriate, 95% confidence intervals are also calculated and reported.

Categorical data is summarised using absolute and relative frequency.

The hypotheses for analysing the mean group differences in the primary endpoint are:

H0: rel-CfB arm A = rel-CfB arm B

versus

H1: rel-CfB Arm A≠ rel-CfB Arm B

This is analysed using an analysis of covariance with the factor treatment group and the covariate baseline. As a sensitivity analysis, the relevant changes are analysed using 2-sample Wilcoxon tests.

The continuous target variables are analysed analogously with the CfBs. The results of these analyses are presented graphically using progression curves (mean values and LS mean changes) of the original mean values and the CfBs. Ordinal endpoints (e.g. PGI) are compared between the groups using 2-sample Wilcoxon tests.
Binary endpoints (rates) are compared using Fisher tests; in addition, 95% CIs are provided for comparison between groups.

Multiplicity: The primary endpoint (1) and the first 3 secondary endpoints (2) - (4) are tested sequentially according to the method of a priori ordered hypotheses: The primary endpoint (1) relative change rel-CfB-IEF week 12 with respect to baseline of incontinence episodes is analysed using an analysis of covariance with the factor treatment group and the baseline value of the IEF as covariate; the significance level is 5% 2-sided. If significance is reached for endpoint (1), endpoint 2 (change in the ICIQ-SF questionnaire) is tested analogue to the 5% 2-sided level. If significance is reached for endpoint (2), endpoint (3) (change in quality of life in the I-QOL questionnaire) is tested 2-sided analogue to the 5% level. If significance is reached for endpoint 3, endpoint 4 (PAM-13) is tested 2-sided analogue to the 5% level. The other endpoints are analysed descriptively with p-values and 95% CIs. The FAS (ITT) is the primary evaluation collective for efficacy; missing values for the main outcome variables (1) - (4) are imputed using the JtR method.

Subgroup analyses:

Descriptive subgroup analyses are performed for the primary and secondary efficacy variables using the ICD code:

- N39.3 Stress incontinence [stress incontinence]
- N39.42 Urge incontinence
- Mixed incontinence - both codes

and using the S/U-IQ (Stress/Urge Incontinence Questionnaire and Classification System) for the indications:

- pure stress urinary incontinence (SUI≥ 4, UUI=0),
- Mixed urinary incontinence, predominantly stress component (SUI >0, SUI > UUI; UUI> 0),
- balanced mixed urinary incontinence (SUI> 0, SUI=UUI),
- Mixed urinary incontinence, predominantly urge component (SUI> 0, UUI> 0, UUI> SUI)
- Pure urge incontinence (UUI≥ 4, SUI =0)

carried out.

Further subgroup analyses are carried out:

- Medication for the treatment of incontinence (yes/no),
- Premenopausal/menopausal/postmenopausal status,
- Age categories< 45, 45-60,> 60 years,
- BMI,
- Country of birth,
- Level of education,
- Secondary diseases,
- Smoking behaviour,
- Alcohol consumption,
- Number of births,

- Type of delivery,
- Time since last delivery
- Duration of illness,
- Pre-existing pelvic floor course,
- Non-bladder-specific medication,
- Frequency of use of the app.

The I-QOL and ICIQ are also analysed individually.

The results of the subgroup analyses are also graphically presented and summarised using forest plots.

Evaluation collectives:

- Full Analysis Set (FAS) contains all patients who were randomised. Patients who withdrew their consent immediately after randomisation are not included in this data set.
- Per-Protocol Set (PPS) contains all FAS patients from whom no significant study protocol violations were reported.
- Complete cases (CCS): Patients with complete data per evaluation.
- Safety Set (SAF) contains all patients who are included in the study (signed informed consent form). It is expected that SAF and FAS are identical.

Sensitivity analyses are carried out:

1. CCS analysis
2. FAS analysis with last-observation-carried-forward-option (LOCF).

8.9 Presentation of the results

The results are presented in close accordance with the guideline "Structure and Content of Clinical Study
Reports E3" (ICH-E3) are presented in tabular form (Section 8.10) and as listings (Section 8.11).

8.10 Tables

- Demographic data and other baseline characteristics
- Primary endpoint
- Secondary endpoints

8.11 Listings

- List of dropouts who have withdrawn their declaration of consent (WoC)
- Curriculum deviations - (NtF)

Docusign Envelope ID: 05A6509E-2636-42E1-8D3B-8B2D12CE3674

- Patients for whom deviations from the study plan lead to exclusion from the PPS (drop-outs)
- Demographic data (height, age, weight, etc.)
- Other baseline characteristics
- Individual data and results for primary and secondary endpoints
- Adverse events (AE/SAE and events)

8.12 Data collection/documentation forms

In order to achieve the study objective, it is necessary to collect and process medical data from individual patients. Data is collected using electronic questionnaires, which are completed by the patients at the beginning (baseline) and at the end of the study (PROM).

The data to be analysed from the patients is collected in the app on the one hand and via an ePRO system on the other, and the latter is stored in an eCRF. The questionnaires, which are to be completed independently at the beginning (baseline/V1) and at the end of the study (V3), are collected via an ePRO system and stored in the EvidentIQ software. Further technical parameters are listed in the separate document "Confirmation of validation of EvidentIQ Germany's Marvin software and secure data hosting".

A detailed statistical analysis plan (SAP) is created before the evaluation.

9 Data management

9.1 Patient identification

All patient-related data is collected by Kranus Health GmbH in pseudonymised form. For this purpose, a non-verbal pseudonym is used, from which alone the identity of the patient cannot be deduced. The patient identification number is assigned centrally as part of the internet-based registration process.

After registration of the patient and scheduling of the informed consent discussion, the trial centre receives an e-mail with the patient identification number and the information that a new registration and scheduling has been received. This e-mail, together with the information provided by the patient during registration, is used by the trial centre to carry out the patient information on behalf of the sponsor and to have the patient information provided by Kranus signed to obtain consent for participation in the trial. Once consent has been obtained, it will be together with the patient identification list for the sponsor for 10 years in accordance with the requirements of the MDR.

9.2 Data acquisition

Electronic data recording is carried out via EvidentIQ's eCRF on a proprietary server. Information on data security and compliance of the server structure is specified in the appendix.

9.3 Data protection

As part of the study, is necessary to collect and process personal data from the study participants (e.g. full name, initials of first name and surname, date of birth, address) and data on treatment and the course of the disease (e.g. medical findings, types of treatment, prescribed medication). This data is generated by the patients themselves and stored electronically on the study server in pseudonymised form (i.e. without direct reference to the patient's name) with the help of a patient identification number, transmitted to the responsible data processing body and evaluated.

The trial participants will be informed about the disclosure of their pseudonymised data to the recipients named therein as part of the documentation and notification obligations. Persons who do not consent to the disclosure will not be included in the clinical trial.

If patients withdraw their consent to the study, including further data collection, no further data will be collected from the time of withdrawal. The data collected to date will continue to be used and analysed within the study, but can be deleted at the patient's request during the study period. If a patient only cancels the study treatment, the data required for the study can continue to be collected and used as long as the patient has not withdrawn their consent.

9.4 Quality assurance

Only PROMs are collected in the study; monitoring is not planned.

9.5 Archiving

All documentation forms, consent forms and other important trial documents are stored for at least 10 years in accordance with Section 13 (10) GCP-V. The patient identification list is kept separately from the documentation records in all centres.

10 Ethical issues, legal and administrative regulations

10.1 Declaration of Helsinki and Good Clinical Practice

The study is conducted in accordance with the ethical principles that have their origin in the Declaration of Helsinki [39]. The current version of the Declaration will be observed.

The recommendations of Good Clinical Practice [39], valid since 17 January 1997, are taken into account where applicable.

10.2 Independent ethics committees

The study protocol is submitted with the necessary additional documents to the responsible ethics committee of the scientific director with a request for evaluation. The study can only begin after the ethics committee has given its approval.

10.3 Legal regulations

The Kranus Mictera app is an approved Class I medical device.

10.4 Registration

The study should be entered in the following public register: German Clinical Trials Register (DKRS, https://www.drks.de).

10.5 Financing

The study is financed by Kranus Health GmbH.

10.6 Final report and publication

The final report will be completed by 15 April 2025. Publication will be primarily in a peer-reviewed journal. The order of authors will be determined by the scientific director in collaboration with the sponsor. The results of the study will be published regardless of the outcome.

11 Bibliography

1. Beutel, M.E., et al, *[Prevalence of urinary incontinence in the German population].* Urologist A, 2005. **44**(3): p. 232-8.
2. DGGG, O., SGGG *Female Urinary Incontinence. Guideline of the DGGG, OEGGG and SGGG (S2k- Level, AMWF Registry No. 015-91, December 2021*. 2021.
3. Burkhard, F.C., et al, *EAU guidelines on Urinary Incontinence in Adults*. 2020, EAU Guideline Office: Arnhem.
4. Todhunter-Brown, A., et al, *Conservative interventions for treating urinary incontinence in women: an Overview of Cochrane systematic reviews.* Cochrane Database Syst Rev, 2022. **9**(9): p. CD012337.
5. Dumoulin, C., et al, *Pelvic-Floor-Muscle Training Adherence: Tools, Measurements and Strategies-2011 ICS State-of-the-Science Seminar Research Paper II of IV.* Neurourol Urodyn, 2015. **34**(7): p. 615-21.
6. Yalcin, I., et al, *Reductions in stress urinary incontinence episodes: what is clinically important for women?* Neurourol Urodyn, 2010. **29**(3): p. 344-7.
7. Shamliyan, T., J. Wyman, and R.L. Kane, *AHRQ Comparative Effectiveness Reviews*, in *Nonsurgical Treatments for Urinary Incontinence in Adult Women: Diagnosis and Comparative Effectiveness*. 2012, Agency for Healthcare Research and Quality (US): Rockville (MD).
8. Asklund, I., et al, *Mobile app for treatment of stress urinary incontinence: A randomised controlled trial.* Neurourol Urodyn, 2017. **36**(5): p. 1369-1376.
9. Yalcin, I., et al, *Minimal clinically important differences in incontinence quality-of-life scores in stress urinary incontinence.* Urology, 2006. **67**(6): p. 1304-8.
10. Minassian, V.A., H.P. Drutz, and A. Al-Badr, *Urinary incontinence as a worldwide problem.* Int J Gynaecol Obstet, 2003. **82**(3): p. 327-38.
11. Valdevenito, J.P., et al, *Voiding symptoms obtained by open versus directed anamnesis as predictors of voiding dysfunction in women.* Int Braz J Urol, 2019. **45**(4): p. 798-806.
12. Elmer, C., et al, *Twenty-Four-Hour Voiding Diaries Versus 3-Day Voiding Diaries: A Clinical Comparison.* Female Pelvic Med Reconstr Surg, 2017. **23**(6): p. 429-432.
13. Painter, V., E. Karantanis, and K.H. Moore, *Does patient activity level affect 24-hr pad test results in stress-incontinent women?* Neurourol Urodyn, 2012. **31**(1): p. 143-7.
14. Tseng, L.H., et al, *Postvoid residual urine in women with stress incontinence.* Neurourol Urodyn, 2008. **27**(1): p. 48-51.
15. Cacciari, L.P., C. Dumoulin, and E.J. Hay-Smith, *Pelvic floor muscle training versus no treatment, or inactive control treatments, for urinary incontinence in women: a cochrane systematic review abridged republication.* Braz J Phys Ther, 2019. **23**(2): p. 93-107.
16. Hunskaar, S., *A systematic review of overweight and obesity as risk factors and targets for clinical intervention for urinary incontinence in women.* Neurourol Urodyn, 2008. **27**(8): p. 749- 57.
17. Radzimińska, A., et al, *The impact of pelvic floor muscle training on the quality of life of women with urinary incontinence: a systematic literature review.* Clin Interv Aging, 2018. **13**: p. 957- 965.
18. Paiva, L.L., et al, *Pelvic floor muscle training in groups versus individual or home treatment of women with urinary incontinence: systematic review and meta-analysis.* Int Urogynecol J, 2017. **28**(3): p. 351-359.
19. Hay-Smith, E.J., et al, *Comparisons of approaches to pelvic floor muscle training for urinary incontinence in women.* Cochrane Database Syst Rev, 2011(12): p. Cd009508.
20. Berghmans, B., et al, *Efficacy of physical therapeutic modalities in women with proven bladder overactivity.* Eur Urol, 2002. **41**(6): p. 581-7.

Docusign Envelope ID: 05A6509E-2636-42E1-8D3B-8B2D12CE3674

21. Boyle, R., et al, *Pelvic floor muscle training for prevention and treatment of urinary and faecal incontinence in antenatal and postnatal women.* Cochrane Database Syst Rev, 2012. **10**: p. CD007471.
22. Kim, H., et al, *Effectiveness of multidimensional exercises for the treatment of stress urinary incontinence in elderly community-dwelling Japanese women: a randomised, controlled, crossover trial.* J Am Geriatr Soc, 2007. **55**(12): p. 1932-9.
23. McFall, S.L., A.M. Yerkes, and L.D. Cowan, *Outcomes of a small group educational intervention for urinary incontinence: health-related quality of life.* J Aging Health, 2000. **12**(3): p. 301-17.
24. Cipullo, L.M., et al, *Pharmacological approach to overactive bladder and urge urinary incontinence in women: an overview.* Eur J Obstet Gynecol Reprod Biol, 2014. **174**: p. 27-34.
25. Chapple, C.R., et al, *Mirabegron in overactive bladder: a review of efficacy, safety, and tolerability.* Neurourol Urodyn, 2014. **33**(1): p. 17-30.
26. Loohuis, A.M.M., et al, *App-Based Treatment in Primary Care for Urinary Incontinence: A Pragmatic, Randomised Controlled Trial.* Ann Fam Med, 2021. **19**(2): p. 102-109.
27. Hou, Y., et al, *Effect of pelvic floor muscle training using mobile health applications for stress urinary incontinence in women: a systematic review.* BMC Womens Health, 2022. **22**(1): p. 400.
28. Bjelic-Radisic, V., et al, *Psychometric properties and validation of two global impression questionnaires (PGI-S, PGI-I) for stress incontinence in a German-speaking female population.* Neurourol Urodyn, 2018. **37**(4): p. 1365-1371.
29. Bent, A.E., et al, *Validation of a two-item quantitative questionnaire for the triage of women with urinary incontinence.* Obstet Gynecol, 2005. **106**(4): p. 767-73.
30. Dumoulin, C., L.P. Cacciari, and E.J.C. Hay-Smith, *Pelvic floor muscle training versus no treatment, or inactive control treatments, for urinary incontinence in women.* Cochrane Database Syst Rev, 2018. **10**(10): p. Cd005654.
31. Shamliyan T, W.J., Kane RL, *Nonsurgical Treatments for Urinary Incontinence in Adult Women: Diagnosis and Comparative Effectiveness [Internet].* in *Comparative Effectiveness Reviews*. 2012, Agency for Healthcare Research and Quality (US): Rockville (MD.
32. Lim, R., et al, *Which outcome measures should be used in stress urinary incontinence trials?* BJU Int, 2018. **121**(5): p. 805-810.
33. Sjöström, M., et al, *Internet-based treatment of stress urinary incontinence: a randomised controlled study with focus on pelvic floor muscle training.* BJU Int, 2013. **112**(3): p. 362-72.
34. Bø, K., T. Talseth, and I. Holme, *Single blind, randomised controlled trial of pelvic floor exercises, electrical stimulation, vaginal cones, and no treatment in management of genuine stress incontinence in women.* Bmj, 1999. **318**(7182): p. 487-93.
35. Castro, R.A., et al, *Single-blind, randomised, controlled trial of pelvic floor muscle training, electrical stimulation, vaginal cones, and no active treatment in the management of stress urinary incontinence.* Clinics (Sao Paulo), 2008. **63**(4): p. 465-72.
36. Avery, K., et al, *ICIQ: a brief and robust measure for evaluating the symptoms and impact of urinary incontinence.* Neurourol Urodyn, 2004. **23**(4): p. 322-30.
37. Nyström, E., L. Söderström, and E. Samuelsson, *Self-management of incontinence using a free mobile app: factors associated with improvement.* Int Urogynecol J, 2022. **33**(4): p. 877-885.
38. Nystrom, E., et al, *ICIQ symptom and quality of life instruments measure clinically relevant improvements in women with stress urinary incontinence.* Neurourol Urodyn, 2015. **34**(8): p. 747-51.
39. Association, W.M., *WMA Declaration of Helsinki - Ethical Principles for Medical Research Involving Human Subjects*. 2013, World Medical Association: Fortaleza (Brazil).

Docusign Envelope ID: 05A6509E-2636-42E1-8D3B-8B2D12CE3674

A. Appendix

A1. Questionnaires used

A1.1 PGI-I: Patient Global Impressions Scale - Improvement

**Please select the number that best reflects the condition of your incontinence now in the compared to before the therapy:**	
Much better	1
Much better	2
A little better	3
unchanged	4
A little worse	5
Much worse	6
Much worse	7

A1.2 I-QOL: Urinary Incontinence Quality of Life Scale

Questions	Possible answers
1 I'm afraid I won't be able to reach the toilet in time.	**1** very<br>**2** quite<br>**3** moderate<br>**4** a little<br>**5** not at all
2 I am afraid to cough or sneeze because of my urinary problems or incontinence.	**1** very<br>**2** quite<br>**3** moderate<br>**4** a little<br>**5** not at all
3. because of my urinary problems or incontinence, I have to be careful when I get up from a sitting position.	**1** very<br>**2** quite<br>**3** moderate<br>**4** a little<br>**5** not at all
4 I am worried about where toilets are in unknown places.	**1** very<br>**2** quite<br>**3** moderate<br>**4** a little<br>**5** not at all
5 I feel depressed because of my urinary problems or incontinence.	**1** very<br>**2** quite<br>**3** moderate<br>**4** a little<br>**5** not at all
6. because of my urinary problems or incontinence, I don't like to leave the house for long periods of time.	**1** very<br>**2** quite<br>**3** moderate<br>**4** a little<br>**5** not at all
7 I feel frustrated because my urinary problems or incontinence prevent me from doing what I want.	**1** very<br>**2** quite<br>**3** moderate<br>**4** a little<br>**5** not at all
8. i am afraid that others will smell urine on me.	**1** very<br>**2** quite<br>**3** moderate<br>**4** a little<br>**5** not at all
9 My urinary problems or incontinence are constantly on my mind.	**1** very<br>**2** quite<br>**3** moderate<br>**4** a little<br>**5** not at all
10. it is important for me to be able to go to the toilet frequently.	**1** very<br>**2** quite<br>**3** moderate

	**4** a little<br>**5** not at all
11. because of my urinary problems or incontinence, it is important to plan every little thing in advance.	**1** very<br>**2** quite<br>**3** moderate<br>**4** a little<br>**5** not at all
12 I am afraid that my urinary problems or incontinence could get worse as I get older.	**1** very<br>**2** quite<br>**3** moderate<br>**4** a little<br>**5** not at all
13 I have problems sleeping well at night because of my urinary problems or incontinence.	**1** very<br>**2** quite<br>**3** moderate<br>**4** a little<br>**5** not at all
14 I am afraid of being put in an embarrassing or humiliating position because of my urinary problems or incontinence.	**1** very<br>**2** quite<br>**3** moderate<br>**4** a little<br>**5** not at all
15 I don't feel like a healthy person because of my urinary problems or incontinence.	**1** very<br>**2** quite<br>**3** moderate<br>**4** a little<br>**5** not at all
16 My urinary problems or incontinence make me feel helpless.	**1** very<br>**2** quite<br>**3** moderate<br>**4** a little<br>**5** not at all
17 I enjoy life less because of my urinary problems or incontinence.	**1** very<br>**2** quite<br>**3** moderate<br>**4** a little<br>**5** not at all
18 I'm afraid of wetting myself.	**1** very<br>**2** quite<br>**3** moderate<br>**4** a little<br>**5** not at all
19 I have the feeling that I can't control my bladder.	**1** very<br>**2** quite<br>**3** moderate<br>**4** a little<br>**5** not at all
20. because of my urinary problems or incontinence, I have to be careful about what or how much I<br>drink.	**1** very<br>**2** quite<br>**3** moderate

Docusign Envelope ID: 05A6509E-2636-42E1-8D3B-8B2D12CE3674

	**4** a little<br>**5** not at all
21 Because of my urinary problems or incontinence, I am restricted in my choice of clothing.	**1** very<br>**2** quite<br>**3** moderate<br>**4** a little<br>**5** not at all
22 I am afraid to have sex because of my urinary problems or incontinence.	**1** very<br>**2** quite<br>**3** moderate<br>**4** a little<br>**5** not at all

I-QOL: About yourself

A-1 How long have you suffered from bladder problems or

incontinence? (Please enter the number of years and months

below)

A-2 How often have you seen a doctor or undergone physiotherapy for your bladder problems or incontinence in the last year?

(Please enter the number in the space provided)

A-3 How would you describe the severity of your bladder problems or incontinence? (Please circle the number before your answer)

1 MILD 2 MODERATE 3 SEVERE

A-4 Do you leak urine when you cough, sneeze, run, walk, jump or during certain other activities?

0 NO 1 YES

A-5 Do you lose control of your bladder before reach the toilet? 0 NO 1 YES

A-6 Do you leak urine without it being related to any particular activity or without feeling the urge to go to the toilet?

0 NO 1 YES

A-8 How often have you involuntarily lost urine, even small amounts, in the last month?

0 NOT AT ALL IN THE LAST MONTH

1 1 TO 2 TIMES IN THE LAST MONTH

2 4 TIMES (ABOUT ONCE A WEEK)

3 2 TO 3 TIMES A WEEK

4 ABOUT ONCE A DAY

5 1 OR 2 TIMES A DAY

6 3 OR 4 TIMES A DAY

7 5 TIMES A DAY OR MORE

A1.3 ICIQ-SF: International Consultation on Incontinence Questionnaire-Urinary Incontinence Short Form

Question	Possible answers
1. **How often lose you urine?**	**0** never<br>**1** about once a week or less<br>**2** Two or three times a week<br>**3** about once a day<br>**4** several times a day<br>**5** constantly
2. **we would like to know how much urine you think you lose. How much urine do you usually leak?** (regardless of whether you wear pads or not)	**0** no urine loss<br>**2** a small amount of urine<br>**4** a medium amount of urine<br>**6** a large amount of urine
**3. how much does urine loss generally affect your everyday life?** *Please tick a number between 0 (not at all) and 10*<br>*(a serious problem)*	0 (not at all) 1 2 3 4 5 6 7 8 9 10 (serious problem)
**4. when lose you urine?** *(Please tick all boxes that apply)*	☐ Never - no urine loss<br>☐ Urine leakage before reaching the toilet<br>☐ Leakage of urine when coughing or sneezing<br>☐ Urine leakage during sleep<br>☐ Urine loss during physical activity / sporting activity<br>☐ Leakage of urine after urination and re-dressing<br>☐ Urine leakage without recognisable cause<br>☐ Constant loss of urine

Result: Sum score of questions 1+2+3= ICIQ-SF Score

A1.4 PAM-13: Patient Activation Measure

Questions	Possible answers
1. at the end of the day, I am the one who is responsible for looking after my health.	1 Not true<br>2 Hardly true<br>3 Rather true<br>4 Exactly right
2. the most important thing for my health is to take an active role in my healthcare.	1 Not true<br>2 Hardly true<br>3 Rather true<br>4 Exactly right
3. i am convinced that i can do something myself to prevent illness.	1 Not true<br>2 Hardly true<br>3 Rather true<br>4 Exactly right
4 I know why I am taking each of my medicines.	1 Not true<br>2 Hardly true<br>3 Rather true<br>4 Exactly right
5 I am confident that I know when I need to see a doctor and when I can treat a health problem myself.	1 Not true<br>2 Hardly true<br>3 Rather true<br>4 Exactly right
6 I am confident that I can tell my GP about my concerns, even if he doesn't address them directly.	1 Not true<br>2 Hardly true<br>3 Rather true<br>4 Exactly right
7 I am convinced that I can carry out the necessary medical treatments at home.	1 Not true<br>2 Hardly true<br>3 Rather true<br>4 Exactly right
8 I know the causes of my complaints.	1 Not true<br>2 Hardly true<br>3 Rather true<br>4 Exactly right
9 I know different treatment options for my illnesses.	1 Not true<br>2 Hardly true<br>3 Rather true<br>4 Exactly right
10. i have been able to maintain lifestyle changes such as healthy eating and exercise.	1 Not true<br>2 Hardly true<br>3 Rather true<br>4 Exactly right
11 I know how I can prevent my health from deteriorating.	1 Not true<br>2 Hardly true<br>3 Rather true<br>4 Exactly right
12 I am convinced that I will find solutions if my health deteriorates.	1 Not true<br>2 Hardly true<br>3 Rather true<br>4 Exactly right

Docusign Envelope ID: 05A6509E-2636-42E1-8D3B-8B2D12CE3674

13 I am convinced that I can make changes my lifestyle habits - such as diet and Physical exercise - even in stressful situations times.	1 Not true<br>2 Hardly true<br>3 Rather true<br>4 Exactly right

Docusign Envelope ID: 05A6509E-2636-42E1-8D3B-8B2D12CE3674

A1.5 S/U-IQ: Stress/Urge Incontinence Questionnaire and Classification System

**Question**	**Number of events per week**
1 (SUI): In the last seven days, how many times have you accidentally touched your hands during an activity such as coughing, sneezing, laughing, running, exercising or lifting? Lost urine?	
2 (UUI): In the last seven days, how often have you felt the urge to urinate so suddenly that you could not reach the toilet in time? and have lost urine?	

SUI: Stress urinary incontinence episodes per week UUI:
Urge urinary incontinence episodes per week

CLASSIFICATION SYSTEM FOR INCONTINENCE SYMPTOMS WITH S/U-IQ:
- SUI≥ 4; UUI= 0: pure stress urinary incontinence
- SUI> 0; SUI> UUI; UUI> 0: mixed urinary incontinence, predominantly stress component
- SUI> 0; SUI= UUI: balanced mixed urinary incontinence
- SUI> 0; UUI> 0; UUI> SUI: mixed urinary incontinence, predominant urge component
- UUI≥ 4; SUI= 0: pure urge urinary incontinence
