# Supplementary material for: Long-term effectiveness of app-based therapy for female urinary incontinence: 48-week follow-up of a randomised controlled trial
Source: eClinicalMedicine. 2026 Oct 28;101:104298. doi: 10.1016/j.eclinm.2026.104298 (PMC13735171; doi:10.1016/j.eclinm.2026.104298)
Supplement: Supplementary file 2 [file mmc2.pdf]

Docusign Envelope ID: 90D9ED51-3780-4E44-982C-B1C6C4E6D459

Statistical analysis plan

Prospective randomised study on multimodal self-treatment of women with incontinence complaints using a digital health application

(Digital incontinence study, EudraCT-No.: 2024-512094-27-00; DRKS ID: DRKS00033704; ClinicalTrials.gov study ID: NCT06389838)

Study protocol:

Version	date	Version Description
1.0	05 March 2024	First version
2.1	14 November 2024	Final version

This document is a translated version of the original German version. While every effort has been made to ensure accuracy, minor language discrepancies may occur.

SAP:

Version	date	Author	Description
Draft 1.0	12 April 2024	Michael Bulitta	Initial version
Draft 1.1	24 April 2024	Michael Bulitta	Revised version Final
Final version 2.0	14 November 2024	Michael Bulitta	version

This document may not be published, reproduced or forwarded without the written authorisation of Kranus Health GmbH.

Docusign Envelope ID: 90D9ED51-3780-4E44-982C-B1C6C4E6D459

SAP authorisation

SAP Author:

DocuSigned by: Dipl.Stat. Michael Bulitta

26.11.2024

Dipl.-Stat. Michael Bulitta
CRM Biometrics GmbH
Mary-Anderson-Str. 6
53332 Bornheim

Date

SAP Reviewer:

DocuSigned by:

26.11.2024

M. Sc. Daniel Schmitz
CRM Biometrics GmbH
Mary-Anderson-Str. 6
53332 Bornheim

Date

Reviewed and approved by: Project Manager:

DocuSigned by:
92B1BA59491045F...

26.11.2024

Dr Laura Elisa Wiemer
Kranus Health GmbH
Westenrieder Str. 10
80331 Munich

Date

Docusign Envelope ID: 90D9ED51-3780-4E44-982C-B1C6C4E6D459

TABLE OF CONTENTS

STATISTICAL ANALYSIS PLAN 1

1 INTRODUCTION 7

2 DESIGN OF THE STUDY 7

3 SCHEDULE 7

4 STUDY OBJECTIVES 7

4.1.1 Primary goal 7
4.1.2 Secondary objectives 7

5 VARIABLES 8

5.1 Effectiveness variables 8
5.1.1 Primary efficacy variable 8
5.1.2 Secondary effectiveness variables 8
5.2 Compatibility variables 8
5.3 Other variables 9
5.4 Derived variables 9
5.5 Business round scheme 9

6 SAMPLE SIZE 11

7 DATA MANAGEMENT 11

8 COMPUTER SYSTEMS, SOFTWARE, AND PROGRAMME VALIDATION 11

8.1 Hardware 11
8.2 Software 11
8.3 Validation of the programmes 12
8.4 Restitution of the programmes 12

9 DATA-CHECKS 12

10 DATA REVIEW MEETINGS 13

11 STATISTICAL EVALUATION 13

11.1 General information 13
11.2 Evaluation collectives 13
11.3 Patient disposition and characteristics 13
11.4 Effectiveness 14
11.4.1 Primary efficacy variable 14
11.4.2 Secondary effectiveness variables 14
11.5 Compatibility 14
11.6 Statistical analysis 14
11.6.1 Effectiveness 14
11.6.2 Compatibility 15
11.7 Multiplicity 15
11.8 Missing values 16
11.9 Interim evaluation 16
11.10 Patient disposition 16
11.11 Exposure duration 16
11.12 Subgroup analyses 16

Docusign Envelope ID: 90D9ED51-3780-4E44-982C-B1C6C4E6D459

12 VISIT WINDOW 17

13 LANGUAGE AND HEADLINES 17

14 ARCHIVING 17

15 LIST OF EMPLOYEES 18

16 SOURCES 18

Abbreviations

Abbreviations	Explanation
AE	Adverse Event
Fig.	Illustration
AK	Exclusion criteria
AWMF	Association of the Scientific Medical Societies in Germany
BfArM	Federal Institute for Drugs and Medical Devices BMI<br>Body Mass Index
CCS	Complete cases
CfB	Change from Baseline
d	Day - Day(s)
DiGA	Digital health application
DINKS	Digital Incontinence Study (study acronym)
eCRF	electronic Case Report Form
ePRO	electronic Patient-Reported-Outcomes
EK	Inclusion criteria
EMS	Electrical muscle stimulation
FAS	Full Analysis Set
FPI	First Patient In
FPO	First Patient Out
GCP	Good Clinical Practice
ICD	International Classification of Diseases
ICH-E3	Structure and Content of Clinical Study Reports E3
ICH-GCP	International Conference on Harmonisation - Good Clinical<br>Practice Guidelines
ICIQ-SF	Incontinence Questionnaire-Urinary Incontinence Short Form I-
QOL	Incontinence quality of life questionnaire
IEF	Incontinence episode frequency
ISO	International Organisation for Standardization
JtR	Jump-to-Reference
CI	Confidence interval
LOCF	Last Observation Car Ried Forward
LPI	Last Patient In
LPO	Last Patient Out
n. a.	Not applicable
NN	Nomen nominandum
NtF	Note to File
MDR	Medical Device Regulation
MID	Minimum Important Difference
OAB	Overactive Bladder
PAM-13	Patient Activation Measure 13
PGI-I	Patient Global Impression of Improvement -Incontinence
PMR	Progressive Muscle Relaxation
PPS	Per Protocol Set
Rel-CfB	Relative Change from Baseline (CfB/Baseline)
PROM	Patient reported outcome measures
QOL	Quality of Life
SAE	Serious Adverse Event
SAP	Statistical analysis plan
SAF	Safety Set

SF	Short Form
S/UIQ	Stress/Urge Incontinence Questionnaire
V	Visit
vs.	Versus
WoC	Withrawal of consent

1 INTRODUCTION

General information on the methods and procedures of the statistical analysis are described in the final study protocol, version 1.0_final, dated 5 March 2024. This Statistical Analysis Plan (SAP) specifies the analyses of the final statistical analysis and contains details on the endpoints, statistical methods and presentation of the results. This SAP contains extensive and detailed information on the statistical methods of the study protocol.

2 DESIGN OF THE STUDY

This study is designed as a randomised, controlled, two-arm, single-blind, monocentric study. Study arms are:

- Arm A: Intervention through digital therapy (intervention group; multimodal self-treatment of incontinence),
- Arm B: No access to digital therapy, access is granted after the end of the study (control group; waiting group design).

The duration of the intervention is 12 weeks.

It is planned to enrol 198 patients (arm A to arm B in a 1:1 ratio) with stress, urge or mixed incontinence.

3 SCHEDULE

The treatment phase of the study consists of three appointments: V1 (baseline) in week 1, V2 in week 6 (by telephone) and V3 in week 12. There are also two further "follow-up" appointments (outside the study period) after 24 and 36 weeks.

A detailed test scheme is presented in chapter 5.5.

4 STUDY OBJECTIVES

4.1.1 Primary goal

The primary objective of the study is the:

- Reduction in the frequency of incontinence episodes (IEF).

4.1.2 Secondary goals

Secondary objectives of the study are:

- Improvement of disease symptoms in the ICIQ-SF,
- Improving the quality of life in the I-QOL,
- Improvement in the PAM-13 questionnaire (patient activation),
- Superiority in the PGI-I questionnaire (overall improvement after treatment),
- Increase in the proportion of patients without incontinence (cured patients),
- Reduction in micturition frequency during the day and at night,

Docusign Envelope ID: 90D9ED51-3780-4E44-982C-B1C6C4E6D459

- Reduction in pad consumption (pad use),
- Reduction of the imperative urge to urinate,
- Improvement of functional bladder capacity,
- Reduction in body weight,
- Reduction in the proportion of patients with treatment failure,
- Increase in the proportion of patients with a reduction in IEF from baseline to week 12 by at least 50 %,
- Reduction in the frequency of stress incontinence episodes in the S/U-IQ,
- Reduction in the frequency of urge incontinence episodes in the S/U-IQ.

5 VARIABLES

5.1 Effectiveness variables

5.1.1 Primary efficacy variable

Primary efficacy variable is the relative change (rel-CfB-IEF) in IEF from baseline to week 12 relative to baseline; desired reduction in mean relative IEF by 50 % [6] [7]; IEF per 24 hours - averaged over 72 hours.

5.1.2 Secondary effectiveness variables

Secondary efficacy variables (secondary objective criteria) are:

- Change (CfB) in the ICIQ-SF from baseline to week 12; expected mean difference between the groups of > 2.9 points [8, 9],
- Change in I-QoL from baseline to week 12; expected mean difference between the groups of > 6.3 points [9],
- Change in the PAM-13 questionnaire from baseline to week 12,
- PGI-I questionnaire (overall improvement after treatment) at week 12,
- Proportion of patients without incontinence (cured patients) at the end of the study,
- Change in micturition frequency per day from baseline to week 12,
- Change in micturition frequency at night from baseline to week 12,
- Change in pad consumption from baseline to week 12,
- Change in imperative urge to urinate from baseline to week 12,
- Change in functional bladder capacity from baseline to week 12,
- Change body weight from baseline to week 12,
- Proportion of patients with treatment failure within the study period,
- Proportion of patients with a reduction in IEF from baseline to week 12 of at least 50 %,
- Relative change of stress incontinence episodes of the S/U-IQ from the initial situation (baseline) to week 12,
- Relative change in urge incontinence episodes of the S/U-IQ from baseline to week 12.

5.2 Compatibility variables

Compatibility variables are:

- Adverse events (AEs),
- Serious adverse events (SAEs).

5.3 Other variables

- Demographic variables and other baseline characteristics.

5.4 Derived variables

The absolute changes (change from baseline /cfb) are calculated for quantitative variables. Baseline is date 1.

5.5 Business round scheme

The following table shows the ward round schedule of the study.

Docusign Envelope ID: 90D9ED51-3780-4E44-982C-B1C6C4E6D459

Table 1: Visiting schedule

			Study implementation/treatment phase					Follow-up	
	Before enrolment/ Pre-screening	Inclusion	Baseline/ telephone V1 Week 1*	Onboarding Intervention group/ after graduation	Telephone V2 Week 6*	End of study Telephone V3 Week 12*	Onboarding Control group patients	Questionnaire Week 24*	Questionnaire Week 36*
								Both patient groups	
Medical history/ basic data collection	X	X							
Stress-Urge-Incontinence-questionnaire (S/U-IQ)			X			X		X	X
Review Inclusion/exclusion criteria	X	X							
Informed consent/declaration of consent		X							
Randomisation			X						
Documentation of activities in the app				X (Intervention)	X (I)	X (I)	X	X	X
Questionnaires (I-QOL and ICIQ-UI-SF)			X			X		X	X
PGI-I						X		X	X
Technical support contact				X	X	X	X	X	
Micturition diary				X (continuous documentation possible)	X	X	X	X	X
Incontinence documentation over 72 h		X		X (continuous documentation possible)	X	X	X	X	X
Recording of (new) concomitant medications/ Therapies	X	X	X		X	X	X	X	X
Recording of adverse events		X	X	X	X	X	X	X	X

*Week ±7 d

**IAU/SAE/Incidentes via SZ, by telephone (Tel: +49 89 380 38658) or via Mai study-safety@kranus.de

+

6 SAMPLE SIZE

Planning the scope of the study for comparisons between the groups:

- IEF: Relative reduction in incontinence episodes (rel-CfB-IEF) with desired improvement of 50%, Minimum Important Difference (MID) approx. 50% from Yalcin 2010; at d/s= 0.7 with significance level α= 5% 2-sided and Power 1 - β= 90%: N= 2 x 44 = 88, + 20% due to dropouts and their conservative imputation as well as subgroup analyses: N = 120.
- ICIQ-SF: CfB with desired improvement by d = 2.9 vs. control (MID = 2.5 from Nyström 2014 and Asklund 2017) by d/s= 2.9/3.2= 0.9 with α= 5% 2-sided and Power 1 - β = 90%: N = 2 x 28.
- I-QoL: CfB with desired improvement by 6.3 vs. control (MID= 6.3 from Yalcin 2006) by d/s = 6.3/ 14 = 0.45 (with α = 5% 2-sided and power = 80%: N = 2 x 90.
- PAM-13: CfB with desired improvement by 6 points vs. control (MCID = 6, i.e. approx. 15% of the PAM rank from 13 - 52 and from John, 2020) by d/s = 6/12 = 0.5 (with α = 5% 2-sided and power = 80%: N = 2 x 64.

Due to dropouts and their conservative imputation, the total number of cases is set at 2 x 99 study participants for the evaluation of the primary endpoints.

7 DATA MANAGEMENT

The data management and query creation is carried out by Kranus Health GmbH and the CRO CW-Research & Management GmbH, D-81829 Munich, Konrad-Zuse-Platz 8.

8 COMPUTER SYSTEMS, SOFTWARE, AND PROGRAMME VALIDATION

8.1 Hardware

The following hardware is used at CRM Biometrics GmbH:

- Network servers:
- TERRA SERVER 6530 G3, 946 GB SSD, 16 GB Ram.
- Desktops (workstations):
  6 desktops based on AMD and Intel
- Scanners:
  F-Secure Antivirus 7.0 for Windows Server
  ESET NOD 32 Antivirus Version 9 is used on the desktops
- Backup method:
  Continuous backup using VEEAM.
  Every Friday, a full backup is performed using Acronis True Image echo for Windows server (system and data). A differential backup is created during the rest of the week. The backups are stored internally (RAID 5) and on external hard discs.

8.2 Software

The following software is used at CRM Biometrics GmbH:

- SAS Version 9.4,
- Windows Server 2019 Network servers,
- Windows 10 on Workstations,
- Microsoft Office 2019 and Adobe Professional for reports.

The statistical analysis is performed using the validated Statistical Analysis Programme SAS, version 9.4 under Windows 10.

8.3 Validation of the programmes

All SAS programmes start with a header containing the following information:

- Name of the programme,
- Author,
- Creation date,
- analysis data sets used.

All tables, graphics and listings contain the name of the programme and the creation date at the bottom.

All formats are stored in a permanent format catalogue.

All SAS programmes are checked by a second SAS programmer who is responsible for quality control. He/she will also check all LOG files for ERROR or WARNING messages. ERROR messages must not occur. WAR-NING messages are also checked to see whether they lead to incorrect results. Furthermore, the programmer responsible for QC will all tables, figures and listings for completeness and consistency. The check is documented on the programme validation form.

The biometrician responsible for the analysis ensures that all specifications of the planned analysis have been correctly transferred to the SAS programmes.

8.4 Restitution of the programmes

All SAS programmes used in the analysis and the corresponding "outputs" (listings, tables) are stored and archived together with the corresponding source codes of the programmes at CRM Biometrics GmbH.

9 DATA-CHECKS

Initial data checks of contact details and inclusion and exclusion criteria are carried out by Kranus Health GmbH.

10 DATA REVIEW MEETINGS

Data review meetings were held monthly with the study centres and the CRO CW-Research & Management GmbH.

11 STATISTICAL EVALUATION

11.1 General information

All statistical analyses are performed in accordance with the ICH E9 Guideline.

11.2 Evaluation collectives

The following evaluation collectives are defined:

- **Full Analysis Set (FAS)** contains all patients who were randomised. Patients who withdrew their consent immediately after randomisation are not included in this data set.
- **Per-protocol set (PPS)** contains all FAS patients from whom no major study protocol violations (see next paragraph) were reported. Complete cases (CCS): Patients with complete data per analysis.
- **Safety Set (SAF)** contains all patients who are included in the study (signed informed consent). It is expected that SAF and FAS are identical

The following considered study violations:

- Failure to fulfil the inclusion criteria,
- Fulfilment of the exclusion criteria,
- Lack of participation in the surveys/non-completion of the study questionnaires,
- False information in the interviews/medical history/study questionnaires.

Sensitivity analyses are also carried out:

1. Complete Case Analysis (CCS),
2. FAS analysis with last-observation-carried-forward-option (LOCF).

The primary evaluation collective for effectiveness is the FAS population (ITT).

11.3 Patient disposition and characteristics

Demographic variables and other baseline characteristics are presented descriptively for the FAS, PP, and Safety populations (if different), overall and per treatment group. Quantitative variables are presented using means, medians, standard deviations and extremes. For qualitative variables, corresponding absolute and relative frequencies are determined.

Important baseline characteristics are also presented according to gender and age.

Docusign Envelope ID: 90D9ED51-3780-4E44-982C-B1C6C4E6D459

11.4 Effectiveness

The main time point for the target evaluation is after 12 weeks, i.e. the end of the study for intervention arm A and the delayed start of the intervention in control arm B.

11.4.1 Primary efficacy variable

(1) The primary outcome variable is the relative change (rel-CfB-IEF) in IEF from baseline to week 12.

11.4.2 Secondary effectiveness variables

Secondary effectiveness variables are:

(2) Change in the ICIQ-SF from baseline to week 12.
(3) Change in I-QoL from baseline to week 12.
(4) Change in the PAM-13 questionnaire from baseline to week 12.
(5) PGI-I questionnaire (overall improvement after treatment) at the end of the study.
(6) Proportion of patients without incontinence (cured patients) at the end of the study.
(7) Reduction in the frequency of micturition during the day.
(8) Reduction in micturition frequency at night.
(9) Reduction in pad consumption.
(10) Reduction of the imperative urge to urinate.
(11) Change in functional bladder capacity.
(12) Reduction in body weight.
(13) Reduction in the proportion of patients with treatment failure within the study period.
(14) Proportion of patients with a reduction in IEF from baseline to week 12 of at least 50%.
(15) Change in stress incontinence episodes of the S/U-IQ from baseline to week 12.
(16) Change in urge incontinence episodes of the S/U-IQ from baseline to week 12.

11.5 Compatibility

Adverse events (AEs) and serious adverse events (SAEs) are documented using ISO14155.

11.6 Statistical analysis

11.6.1 Effectiveness

In general, continuous data is summarised using descriptive statistics, i.e. with the number of observations, mean, standard deviation, median, minimum, median and maximum. For some variables, such as the primary endpoints, the change from baseline to the end of the study (change from baseline, CfB) is also output and summarised as "post-baseline". Where appropriate, 95% confidence intervals are also calculated and reported.

Docusign Envelope ID: 90D9ED51-3780-4E44-982C-B1C6C4E6D459

Categorical data is summarised using absolute and relative frequency.

The hypotheses for analysing the mean group differences in the primary endpoint are:

$$H_0\text{: rel-CfB Arm A} = \text{rel-CfB Arm B versus}$$

$$H_1\text{: rel-CfB Arm A} \neq \text{rel-CfB Arm B.}$$

This is analysed using an analysis of covariance with the factor treatment group and the covariate baseline. As a sensitivity analysis, the relevant changes are analysed using two-sample Wilcoxon-Mann-Whitney tests.

The continuous target variables are analysed analogously with the CfBs. The results of these analyses are presented graphically using progression curves (mean values and LS mean changes) of the original mean values and the CfBs.

Ordinal endpoints (e.g. PGI) are compared between groups using two-sample Wilcoxon-Mann-Whitney tests.

Binary endpoints (rates) are compared using Fisher tests; in addition, 95% confidence intervals are given for the comparison between the groups.

11.6.2 Compatibility

The compatibility evaluation is carried out using the Safety Population. (SAF). The incidence of all AEs/SAEs is analysed descriptively according to "Body System" and "Preferred term". The incidence of all AEs is also analysed according to severity categories.

11.7 Multiplicity

The primary endpoint (1) and the first 3 secondary endpoints (2) - (4) will be tested sequentially according to the method of a priori ordered hypotheses: The primary endpoint (1) relative change rel-CfB-IEF week 12 with respect to baseline of incontinence episodes is analysed using an analysis of covariance with the factor treatment group and the baseline value of the IEF as covariates; the two-sided significance level is 5%. If significance is reached for endpoint (1), endpoint 2 (change in the ICIQ-SF questionnaire) is tested two-sided analogue to the 5% level. If significance is reached for endpoint
(2), endpoint (3) (change in quality of life in the I-QOL questionnaire) is tested two-sided analogue to the 5% level. If significance is achieved for endpoint 3, endpoint 4 (PAM-13) is tested two-sided analogue to the 5% level. The other endpoints are analysed descriptively with p-values and 95% confidence intervals.

Docusign Envelope ID: 90D9ED51-3780-4E44-982C-B1C6C4E6D459

11.8 Missing values

Missing values of the main target variables (1) - (4) are imputed using the jump-to-reference method (JtR).

Furthermore, missing values are replaced for a sensitivity analysis using the load observation-carried-forward principle (LOCF).

11.9 Interim evaluation

An interim evaluation is not planned.

11.10 Patient disposition

The patient disposition data (number of screened patients and number of randomised and treated patients, number of dropouts, the number of analysis populations and the number of exclusions from the individual evaluation collectives) are presented in tabular and graphical form.

This data is also listed. Study cancellations are listed with the reasons for cancellation. The dates of the "Informed Consent" and the first and last use of the app are also listed.

11.11 Exposure duration

The individual study duration is calculated for each patient according to the following formula:

$$\text{Exposure duration} =_{\text{last visit}} - d_{\text{visit 1}} + 1\text{"},$$

where $dlast_{visit}$ is the date of the last documented visit and $dvisit_1$ is the date of the first visit.

The duration of exposure is analysed using descriptive statistics.

11.12 Subgroup analyses

It are descriptive subgroup analyses for the primary and the secondary efficacy variables using the ICD code:

- N39.3 Stress incontinence,
- N39.42 Urge incontinence,
- Mixed incontinence - both codes.

and using the S/U-IQ (Stress/Urge Incontinence Questionnaire and Classification System) for the indications:

- pure stress urinary incontinence (SUI≥ 4, UUI=0),
- Mixed urinary incontinence, predominantly stress component (SUI > 0, SUI > UUI; UUI > 0),

Docusign Envelope ID: 90D9ED51-3780-4E44-982C-B1C6C4E6D459

- balanced mixed urinary incontinence (SUI> 0, SUI=UUI),
- Mixed urinary incontinence, predominantly urge component (SUI > 0, UUI > 0, UUI> SUI)
- Pure urge incontinence (UUI4, SUI =0)

carried out.
Further subgroup analyses are carried out:

- Medication for the treatment of incontinence,
- Premenopausal/menopausal/postmenopausal status,
- Age categories < 45, 45-60, > 60 years,
- BMI,
- Country of birth,
- Level of education,
- Secondary diseases,
- Smoking behaviour,
- Alcohol consumption,
- Number of births,
- Type of delivery,
- Time since last delivery,
- Duration of illness,
- Pre-existing pelvic floor course,
- Non-bladder-specific medication,
- Frequency of use of the app.

In addition, the I-QOL and ICIQ are analysed individually.

The results of the subgroup analyses are also graphically presented and summarised using forest plots.

12 VISITING WINDOW

All data will be organised and analysed according to the scheduled dates in the study protocol.

**Date**	**1**	**2**	**3**
week	1<br>(±7 d)	6<br>(±7 d)	12<br>(±7 d)

13 LANGUAGE AND HEADLINES

All tables, graphs and listings as well as the statistical report are prepared in German.

14 ARCHIVING

After completion of the analyses and reporting, the following documents are provided by CRM Biometrics GmbH for archiving purposes:

Docusign Envelope ID: 90D9ED51-3780-4E44-982C-B1C6C4E6D459

- SAP (pdf format),
- Statistical report including all tables, graphs and listings (WORD 2010 format and pdf format).

15 EMPLOYEE LIST

A list of employees of CRM Biometrics GmbH can be made available on request.

16 SOURCES

1. Beutel, M.E., et al, *[Prevalence of urinary incontinence in the German population].* Urologist A, 2005. **44**(3): p. 232-8.
2. DGGG, O., SGGG *Female Urinary Incontinence. Guideline of the DGGG, OEGGG and SGGG (S2k-Level, AMWF Registry No. 015-91, December 2021*. 2021.
3. Burkhard, F.C., et al, *EAU guidelines on Urinary Incontinence in Adults*. 2020, EAU Guideline Office: Arnhem.
4. Todhunter-Brown, A., et al, *Conservative interventions for treating urinary incontinence in women: an Overview of Cochrane systematic reviews.* Cochrane Database Syst Rev, 2022. **9**(9): p. CD012337.
5. Dumoulin, C., et al, *Pelvic-Floor-Muscle Training Adherence: Tools, Measurements and Strategies-2011 ICS State-of-the-Science Seminar Research Paper II of IV.* Neurourol Urodyn, 2015. **34**(7): p. 615-21.
6. Yalcin, I., et al, *Reductions in stress urinary incontinence episodes: what is clinically important for women?* Neurourol Urodyn, 2010. **29**(3): p. 344-7.
7. Shamliyan, T., J. Wyman, and R.L. Kane, *AHRQ Comparative Effectiveness Reviews*, in *Nonsurgical Treatments for Urinary Incontinence in Adult Women: Diagnosis and Comparative Effectiveness*. 2012, Agency for Healthcare Research and Quality (US): Rockville (MD).
8. Asklund, I., et al, *Mobile app for treatment of stress urinary incontinence: A randomised controlled trial.* Neurourol Urodyn, 2017. **36**(5): p. 1369-1376.
9. Yalcin, I., et al, *Minimal clinically important differences in incontinence quality-of-life scores in stress urinary incontinence.* Urology, 2006. **67**(6): p. 1304-8.
10. Minassian, V.A., H.P. Drutz, and A. Al-Badr, *Urinary incontinence as a worldwide problem.* Int J Gynaecol Obstet, 2003. **82**(3): p. 327-38.
11. Valdevenito, J.P., et al, *Voiding symptoms obtained by open versus directed anamnesis as predictors of voiding dysfunction in women.* Int Braz J Urol, 2019. **45**(4): p. 798-806.
12. Elmer, C., et al, *Twenty-Four-Hour Voiding Diaries Versus 3-Day Voiding Diaries: A Clinical Comparison.* Female Pelvic Med Reconstr Surg, 2017. **23**(6): p. 429-432.
13. Painter, V., E. Karantanis, and K.H. Moore, *Does patient activity level affect 24-hr pad test results in stress-incontinent women?* Neurourol Urodyn, 2012. **31**(1): p. 143-7.
14. Tseng, L.H., et al, *Postvoid residual urine in women with stress incontinence.* Neurourol Urodyn, 2008. **27**(1): p. 48-51.
15. Cacciari, L.P., C. Dumoulin, and E.J. Hay-Smith, *Pelvic floor muscle training versus no treatment, or inactive control treatments, for urinary incontinence in women: a cochrane systematic review abridged republication.* Braz J Phys Ther, 2019. **23**(2): p. 93-107.

Docusign Envelope ID: 90D9ED51-3780-4E44-982C-B1C6C4E6D459

16. Hunskaar, S., *A systematic review of overweight and obesity as risk factors and targets for clinical intervention for urinary incontinence in women.* Neurourol Urodyn, 2008. **27**(8): p. 749-57.
17. Radzimińska, A., et al, *The impact of pelvic floor muscle training on the quality of life of women with urinary incontinence: a systematic literature review.* Clin Interv Aging, 2018. **13**: p. 957-965.
18. Paiva, L.L., et al, *Pelvic floor muscle training in groups versus individual or home treatment of women with urinary incontinence: systematic review and meta-analysis.* Int Urogynecol J, 2017. **28**(3): p. 351-359.
19. Hay-Smith, E.J., et al, *Comparisons of approaches to pelvic floor muscle training for urinary incontinence in women.* Cochrane Database Syst Rev, 2011(12): p. Cd009508.
20. Berghmans, B., et al, *Efficacy of physical therapeutic modalities in women with proven bladder* 21. Boyle, R., et al, *Pelvic floor muscle training for prevention and treatment of urinary and faecal incontinence in antenatal and postnatal women.* Cochrane Database Syst Rev, 2012. **10**: p. CD007471.
21. Kim, H., et al, *Effectiveness of multidimensional exercises for the treatment of stress urinary incontinence in elderly community-dwelling Japanese women: a randomised, controlled, crossover trial.* J Am Geriatr Soc, 2007. **55**(12): p. 1932-9.
22. McFall, S.L., A.M. Yerkes, and L.D. Cowan, *Outcomes of a small group educational intervention for urinary incontinence: health-related quality of life.* J Aging Health, 2000. **12**(3): p. 301-17.
23. Cipullo, L.M., et al, *Pharmacological approach to overactive bladder and urge urinary incontinence in women: an overview.* Eur J Obstet Gynecol Reprod Biol, 2014. **174**: p. 27-34.
24. Chapple, C.R., et al, *Mirabegron in overactive bladder: a review of efficacy, safety, and tolerability.* Neurourol Urodyn, 2014. **33**(1): p. 17-30.
25. Loohuis, A.M.M., et al, *App-Based Treatment in Primary Care for Urinary Incontinence: A Pragmatic, Randomised Controlled Trial.* Ann Fam Med, 2021. **19**(2): p. 102-109.
26. Hou, Y., et al, *Effect of pelvic floor muscle training using mobile health applications for stress urinary incontinence in women: a systematic review.* BMC Womens Health, 2022. **22**(1): p. 400.
27. Bjelic-Radisic, V., et al, *Psychometric properties and validation of two global impression questionnaires (PGI-S, PGI-I) for stress incontinence in a German-speaking female population.* Neurourol Urodyn, 2018. **37**(4): p. 1365-1371.
28. Bent, A.E., et al, *Validation of a two-item quantitative questionnaire for the triage of women with urinary incontinence.* Obstet Gynecol, 2005. **106**(4): p. 767-73.
29. Dumoulin, C., L.P. Cacciari, and E.J.C. Hay-Smith, *Pelvic floor muscle training versus no treatment, or inactive control treatments, for urinary incontinence in women.* Cochrane Database Syst Rev, 2018. **10**(10): p. Cd005654.
30. Shamliyan T, W.J., Kane RL, *Nonsurgical Treatments for Urinary Incontinence in Adult Women: Diagnosis and Comparative Effectiveness [Internet].* in *Comparative Effectiveness Reviews*. 2012, Agency for Healthcare Research and Quality (US): Rockville (MD.
31. Lim, R., et al, *Which outcome measures should be used in stress urinary incontinence trials?* BJU Int, 2018. **121**(5): p. 805-810.

Docusign Envelope ID: 90D9ED51-3780-4E44-982C-B1C6C4E6D459

32. Sjöström, M., et al, *Internet-based treatment of stress urinary incontinence: a randomised controlled study with focus on pelvic floor muscle training.* BJU Int, 2013. **112**(3): p. 362- 72.
33. Bø, K., T. Talseth, and I. Holme, *Single blind, randomised controlled trial of pelvic floor exercises, electrical stimulation, vaginal cones, and no treatment in management of genuine stress incontinence in women.* Bmj, 1999. **318**(7182): p. 487-93.
34. Castro, R.A., et al, *Single-blind, randomised, controlled trial of pelvic floor muscle training, electrical stimulation, vaginal cones, and no active treatment in the management of stress urinary incontinence.* Clinics (Sao Paulo), 2008. **63**(4): p. 465-72.
35. Avery, K., et al, *ICIQ: a brief and robust measure for evaluating the symptoms and impact of urinary incontinence.* Neurourol Urodyn, 2004. **23**(4): p. 322-30.
36. Nyström, E., L. Söderström, and E. Samuelsson, *Self-management of incontinence using a free mobile app: factors associated with improvement.* Int Urogynecol J, 2022. **33**(4): p. 877-885.
37. Nystrom, E., et al, *ICIQ symptom and quality of life instruments measure clinically relevant improvements in women with stress urinary incontinence.* Neurourol Urodyn, 2015. **34**(8): p. 747-51.
38. Association, W.M., *WMA Declaration of Helsinki - Ethical Principles for Medical Research Involving Human Subjects*. 2013, World Medical Association: Fortaleza (Brazil). *overactivity.* Eur Urol, 2002. **41**(6): p. 581-7.
