# Supplementary material for: Long-term effectiveness of app-based therapy for female urinary incontinence: 48-week follow-up of a randomised controlled trial
Source: eClinicalMedicine. 2026 Oct 28;101:104298. doi: 10.1016/j.eclinm.2026.104298 (PMC13735171; doi:10.1016/j.eclinm.2026.104298)
Supplement: Supplementary file 3 [file mmc3.docx]

Supplementary Material Follow-Up DINKS Study

EudraCT-Nr.: 2024-512094-27-00

DRKS:

DRKS00033704

Clinicaltrials.gov:

NCT06389838

Table of content

[Tables	1](#_Toc2065374743)

[Table A 1 Univariable association between participant characteristics and loss to follow-up	2](#_Toc621477824)

[Table A 2 Association between nonresponder status and dropout across follow-up	2](#_Toc511791692)

[Table A 3 Distribution of urinary incontinence subtype across follow-up assessments	2](#_Toc387708162)

[Table A 4 Age of completers versus participants lost to follow-up, by timepoint	3](#_Toc324869448)

[Table A 5 ICIQ-UI-SF severity categories over time (complete cases, CC)	3](#_Toc1869329091)

[Table A 6 Time-aligned between-group treatment effect by analysis method	3](#_Toc422908692)

[Table A 7 Age distribution by incontinence subtype	4](#_Toc1046524848)

[Table A 8 Combined Within-Group CfB (complete cases, CC)	4](#_Toc1488358633)

[Table A 9 Time-adjusted responder rates at equivalent post-intervention timepoints (Complete Cases)	4](#_Toc1156809348)

[Figures	5](#_Toc2047480706)

[Figure A 1 ICIQ-UI-SF symptom severity distribution over time by treatment group	6](#_Toc182639329)

[Figure A 2 Comparison between analysis methods	6](#_Toc649465944)

[Figure A 3 Heatmap: Active App-Usage in Minutes per Week and Category	8](#_Toc1233106219)

# Tables

## Table A 1 Univariable association between participant characteristics and loss to follow-up

| Predictor | Week 24 OR (95% CI); p | Week 36 OR (95% CI); p | Week 48 OR (95% CI); p |
| --- | --- | --- | --- |
| UI subtype: stress vs mixed | 0.74 (0.33–1.72); 0.475 | 0.34 (0.15–0.77); 0.010 | 0.59 (0.23–1.40); 0.249 |
| UI subtype: urge vs mixed | 1.30 (0.51–3.40); 0.581 | 0.77 (0.29–1.96); 0.581 | 0.94 (0.32–2.66); 0.907 |
| Age (per year) | 0.97 (0.95–1.00); 0.021 | 0.99 (0.97–1.02); 0.614 | 0.98 (0.96–1.01); 0.173 |
| Baseline ICIQ-UI-SF (per point) | 0.99 (0.88–1.11); 0.896 | 1.06 (0.94–1.19); 0.337 | 1.02 (0.90–1.15); 0.766 |
| Baseline I-QoL (per point) | 1.00 (0.98–1.01); 0.543 | 0.99 (0.98–1.00); 0.172 | 0.99 (0.98–1.01); 0.344 |
| Early ICIQ-UI-SF change (per point) | 0.94 (0.84–1.05); 0.241 | 1.02 (0.92–1.13); 0.704 | 1.00 (0.90–1.10); 0.970 |
| Early I-QoL change (per point) | 1.01 (0.99–1.04); 0.148 | 0.99 (0.98–1.01); 0.558 | 1.00 (0.98–1.02); 0.757 |
| App satisfaction (per point) | 1.21 (0.98–1.54); 0.095 | 1.14 (0.95–1.38); 0.171 | 1.04 (0.87–1.23); 0.674 |
| Comorbidity count (per condition) | 1.14 (0.58–2.15); 0.694 | 1.80 (0.95–3.69); 0.084 | 1.06 (0.56–2.20); 0.868 |
| Current smoking (yes vs no) | 2.52 (0.77–8.81); 0.127 | 1.39 (0.42–4.62); 0.577 | 1.59 (0.46–7.35); 0.499 |
| Education: mid vs low* | 0.55 (0.06–4.73); 0.559 | 0.81 (0.09–6.90); 0.832 | 1.52 (0.18–13.02); 0.680 |
| Education: high vs low* | 0.64 (0.07–5.62); 0.668 | 0.60 (0.07–5.29); 0.625 | 3.31 (0.37–29.46); 0.249 |
| Prior physiotherapy, ever (yes vs no) | 0.63 (0.34–1.17); 0.144 | 0.96 (0.53–1.78); 0.908 | 1.06 (0.56–1.99); 0.854 |
| Physiotherapy, last 6 months (yes vs no) | 0.54 (0.24–1.16); 0.128 | 0.87 (0.41–1.77); 0.698 | 1.15 (0.55–2.52); 0.723 |

Loss to follow-up was defined as a missing ICIQ-UI-SF value at the respective visit and modelled by univariable logistic regression (odds ratio >1 indicates higher odds of dropout). "Early change" denotes the time-aligned first 12-week change from baseline (intervention group: week 0 to 12; control group: week 12 to 24). n=194 for baseline characteristics; n=165 for early-change variables and n=164–174 for app satisfaction and comorbidity count, owing to missing data; *n=4 for the “low”group.

## Table A 2 Association between nonresponder status and dropout across follow-up

| Timepoint | Status | Intervention Nonresponder | | | Control Nonresponder | | |
| --- | --- | --- | --- | --- | --- | --- | --- |
|  |  | n  (%) | OR (95%CI) | p-value | n (%) | OR (95%CI) | p-value |
| Week 12 | Completed | 23 (25·0 %) | − | − | − | − | − |
| Week 24 | Completed | 10 (20·4 %) | − | − | 18 (24·7 %) | − | − |
| Week 24 | Lost to follow-up | 13 (30·2 %) | 1·69 (0·66–4·47) | 0·280 | − | − | − |
| Week 36 | Completed | 12 (25·0 %) | − | − | 8 (15·1 %) | − | − |
| Week 36 | Lost to follow-up | 11 (25·0 %) | 1·00 (0·38–2·58) | 1·000 | 10 (50·0 %) | 5·62 (1·8–18·56) | 0·003 |
| Week 48 | Completed | 6 (22·2 %) | − | − | 9 (27·3 %) | − | − |
| Week 48 | Lost to follow-up | 17 (26·2 %) | 1·24 (0·44–3·83) | 0·692 | 9 (22·5 %) | 0·77 (0·26–2·27) | 0·638 |

OR – Odds Ratio; Nonresponders were defined as participants not achieving improvement of ≥2·0-point reduction for ICIQ-UI-SF.

## Table A 3 Distribution of urinary incontinence subtype across follow-up assessments

| Timepoint | Stress UI, n (%) | Urge UI, n (%) | Mixed, n (%) |
| --- | --- | --- | --- |
| Baseline (n=194) | 121 (62·4%) | 43 (22·2%) | 30 (15·5%) |
| Week 12 (n=186) | 117 (62·9%) | 41 (22·0%) | 28 (15·1%) |
| Week 24 (n=122) | 81 (66·4%) | 23 (18·9%) | 18 (14·8%) |
| Week 36 (n=112) | 80 (71·4%) | 20 (17·9%) | 12 (10·7%) |
| Week 48 (n=66) | 46 (69·7%) | 12 (18·2%) | 8 (12·1%) |

Diagnostic categories were based on ICD coding (stress: N39.3; urge: N39.42; mixed).

## Table A 4 Age of completers versus participants lost to follow-up, by timepoint

| Timepoint | Completers, median age (IQR) | n | Dropouts, median age (IQR) | n | p* |
| --- | --- | --- | --- | --- | --- |
| Week 24 | 53·0 (40·2–59·0) | 122 | 46·5 (39·0–53·0) | 72 | 0·018 |
| Week 36 | 50·0 (39·8–57·2) | 112 | 50·0 (40·0–57·0) | 82 | 0·641 |
| Week 48 | 53·5 (41·0–58·0) | 66 | 47·0 (39·0–56·2) | 128 | 0·115 |

Age in years. Dropout defined as a missing ICIQ-UI-SF value at the respective visit. *p from Mann–Whitney U test comparing completers and dropouts at each timepoint.

## Table A 5 ICIQ-UI-SF severity categories over time (complete cases, CC)

| Group | Severity | W0 | W12 | W24 | W36 | W48 |
| --- | --- | --- | --- | --- | --- | --- |
| Early App (IG) | Slight | 0 (0.0%) | 25 (27.2%) | 11 (22.4%) | 16 (33.3%) | 11 (40.7%) |
|  | Moderate | 60 (62.5%) | 56 (60.9%) | 35 (71.4%) | 25 (52.1%) | 13 (48.1%) |
|  | Severe | 36 (37.5%) | 11 (12.0%) | 3 (6.1%) | 7 (14.6%) | 3 (11.1%) |
| Late App (CG) | Slight | 0 (0.0%) | 0 (0.0%) | 8 (11.0%) | 23 (35.9%) | 18 (46.2%) |
|  | Moderate | 57 (58.2%) | 55 (58.5%) | 53 (72.6%) | 36 (56.2%) | 15 (38.5%) |
|  | Severe | 41 (41.8%) | 39 (41.5%) | 12 (16.4%) | 5 (7.8%) | 6 (15.4%) |

ICIQ-UI-SF severity (3-category condensed, Klovning et al. 2009): 0-5 = slight; 6-12 = moderate; 13-21 = severe. Cells show n (%).

## Table A 6 Time-aligned between-group treatment effect by analysis method

|  | MI-MAR (primary, adj. baseline values) | | | MI-MAR adj.  (age + subtype) | | | CC-ANCOVA | | | MMRM | | |
| --- | --- | --- | --- | --- | --- | --- | --- | --- | --- | --- | --- | --- |
| Timepoint | Est. | 95% CI | p | Est. | 95% CI | p | Est. | 95% CI | p | Est. | 95% CI | p |
| *ICIQ-UI-SF* | | | | | | | | | | | | |
| 12 wks post-int. | −0.90 | [−1.83, 0.03] | 0.059 | −0.93 | [−1.87, 0.01] | 0.053 | −1.01 | [−1.97, −0.06] | 0.038 | −1.07 | [−2.04, −0.10] | 0.031 |
| 24 wks post-int. | 0.18 | [−0.81, 1.17] | 0.719 | 0.16 | [−0.85, 1.16] | 0.761 | 1.05 | [−0.08, 2.17] | 0.068 | 0.88 | [−0.21, 1.97] | 0.114 |
| 36 wks post-int. | 0.08 | [−1.09, 1.25] | 0.892 | 0.06 | [−1.12, 1.25] | 0.919 | 0.80 | [−0.72, 2.32] | 0.296 | 0.68 | [−0.50, 1.86] | 0.258 |
| *I-QoL* | | | | | | | | | | | | |
| 12 wks post-int. | 4.89 | [0.34, 9.44] | 0.035 | 4.99 | [0.44, 9.55] | 0.032 | 4.68 | [−0.09, 9.45] | 0.054 | 5.21 | [0.62, 9.81] | 0.026 |
| 24 wks post-int. | 2.91 | [−1.61, 7.44] | 0.207 | 2.96 | [−1.62, 7.54] | 0.205 | 1.65 | [−3.61, 6.92] | 0.535 | 2.40 | [−2.65, 7.45] | 0.351 |
| 36 wks post-int. | 1.50 | [−3.65, 6.64] | 0.568 | 1.54 | [−3.69, 6.77] | 0.563 | −0.54 | [−7.05, 5.97] | 0.870 | −1.54 | [−6.93, 3.85] | 0.575 |

Values are the adjusted mean difference in change from baseline (intervention − control); a negative value for ICIQ-UI-SF and a positive value for I-QoL favour the intervention. Baseline is each group’s own intervention start (intervention group, week 0; control group, week 12). (1) MI-MAR (primary): multiple imputation under missing-at-random, adjusted for baseline; 100 imputations combined by Rubin’s rules. (2) MI-MAR adjusted: additionally adjusted for the randomisation stratification factors, age category (≤45 / 46–60 / >60 years) and incontinence subtype, in both the imputation and analysis models. (3) CC-ANCOVA: complete-case ANCOVA, baseline-adjusted. (4) MMRM: mixed model for repeated measures, no imputation.

## Table A 7 Age distribution by incontinence subtype

| Age category | n | Stress, n (%) | Urge, n (%) | Mixed, n (%) |
| --- | --- | --- | --- | --- |
| ≤45 years | 78 | 53 (67·9%) | 15 (19·2%) | 10 (12·8%) |
| 46–60 years | 84 | 50 (59·5%) | 17 (20·2%) | 17 (20·2%) |
| >60 years | 32 | 18 (56·2%) | 11 (34·4%) | 3 (9·4%) |
| All | 194 | 121 (62·4%) | 43 (22·2%) | 30 (15·5%) |

Percentages are calculated within each age category. The association between age category and incontinence subtype was not statistically significant (Fisher's exact test p=0·26; χ² p=0·23). ICD diagnosis: stress N39.3; urge N39.42; mixed N39.43.

## Table A 8 Combined Within-Group CfB (complete cases, CC)

| Timepoint | n | Mean CfB | SE | Median (IQR) | 95% CI | p |
| --- | --- | --- | --- | --- | --- | --- |
| ICIQ-UI-SF | | | | | | |
| 12 wks (IG+CG) | 165 | -3.68 (3.14) | 0.24 | -3 (-6 to -2) | [-4.16, -3.20] | <0.001 |
| 24 wks (IG+CG) | 113 | -4.09 (3.19) | 0.30 | -4 (-6 to -2) | [-4.68, -3.49] | <0.001 |
| 36 wks (IG+CG) | 87 | -3.97 (3.55) | 0.38 | -4 (-6 to -2) | [-4.72, -3.21] | <0.001 |
| 48 wks (IG only) | 27 | -4.15 (2.54) | 0.49 | -3 (-6 to -2) | [-5.15, -3.14] | <0.001 |
| I-QoL | | | | | | |
| 12 wks (IG+CG) | 165 | 16.99 (17.36) | 1.35 | 13.6 (5.7 to 26.1) | [14.32, 19.66] | <0.001 |
| 24 wks (IG+CG) | 112 | 16.81 (16.51) | 1.56 | 12.5 (5.7 to 27.6) | [13.72, 19.90] | <0.001 |
| 36 wks (IG+CG) | 86 | 17.11 (17.11) | 1.84 | 12.5 (5.1 to 27.3) | [13.44, 20.78] | <0.001 |
| 48 wks (IG only) | 27 | 17.42 (18.10) | 3.48 | 14.8 (9.7 to 23.3) | [10.26, 24.59] | <0.001 |

## Table A 9 Time-adjusted responder rates at equivalent post-intervention timepoints (Complete Cases)

| Timepoint | n | % [95% CI] |
| --- | --- | --- |
| ICIQ-UI-SF | | |
| 12 wks (Early App) | 92 | 75.0% [64.9%, 83.4%] |
| 24 wks (Early App) | 49 | 71.4% [56.7%, 83.4%] |
| 36 wks (Early App) | 48 | 75.0% [60.4%, 86.4%] |
| 48 wks (IG only) | 27 | 85.2% [66.3%, 95.8%] |
| 12 wks (Late App) | 73 | 75.3% [63.9%, 84.7%] |
| 24 wks (Late App) | 64 | 85.9% [75.0%, 93.4%] |
| 36 wks (Late App) | 39 | 76.9% [60.7%, 88.9%] |
| I-QoL | | |
| 12 wks (Early App) | 92 | 78.3% [68.4%, 86.2%] |
| 24 wks (Early App) | 48 | 75.0% [60.4%, 86.4%] |
| 36 wks (Early App) | 48 | 77.1% [62.7%, 88.0%] |
| 48 wks (IG only) | 27 | 77.8% [57.7%, 91.4%] |
| 12 wks (Late App) | 73 | 65.8% [53.7%, 76.5%] |
| 24 wks (Late App) | 64 | 67.2% [54.3%, 78.4%] |
| 36 wks (Late App) | 38 | 71.1% [54.1%, 84.6%] |

ICIQ-SF Responder: CfB ≤ -2.0, I-QoL Responder: CfB ≥ 6.3. CC: Clopper-Pearson 95% CI.

# Figures

Figure A 1 ICIQ-UI-SF symptom severity distribution over time by treatment group

Figure A 2 Comparison between analysis methods

## Figure A 3 Heatmap: Active App-Usage in Minutes per Week and Category
